# Discovery of an ApoE4-targeted small-molecule SirT1 enhancer for the treatment of Alzheimer’s disease

**DOI:** 10.1038/s41598-025-96131-2

**Published:** 2025-04-23

**Authors:** Jesus Campagna, Sujyoti Chandra, Bruce Teter, Whitaker Cohn, Johnny Pham, Young-Sug Kim, Barbara Jagodzinska, Kanagasabai Vadivel, Parvez Alam, Tina Bilousova, Malaney Young, Chris Elias, Juan Marcucci, Ilinca Flacau, Ainsley Jackman, Samar Padder, Dongwook Wi, Chunni Zhu, Patricia Spilman, Michael E Jung, Dale E Bredesen, Varghese John

**Affiliations:** 1 The Drug Discovery Lab, Department of Neurology, David Geffen School of Medicine, 710 Westwood Plaza, Los Angeles, CA 90095 USA; 2 Department of Molecular and Medical Pharmacology, University of California Los Angeles, 650 Charles E. Young Drive, Los Angeles, CA 90095 USA

**Keywords:** Apolipoprotein E4, Sirtuin 1, SirT1, PRMT5, NFYb, SirT1 enhancer, DDL-218, Memory, Chemical biology, Drug discovery, Molecular biology

## Abstract

Decreased expression of sirtuin 1 (SirT1) has been implicated in Alzheimer’s disease (AD), and as we previously reported, is related to transcriptional repression by the major risk factor for sporadic AD, apolipoprotein E4 (ApoE4). Herein we describe the discovery of an orally brain-permeable small-molecule, DDL-218, that enhanced SirT1 in ApoE4-expressing neuronal cells and a murine AD model. DDL-218 increased the transcription factor NFYb resulting in upregulation of PRMT5. Mechanistic and modeling studies show that binding of ApoE4 to the SirT1 gene promoter can be displaced by PRMT5 leading to increased SirT1 transcription. DDL-218 treatment elicited improvement in memory in the AD model, suggesting that DDL-218 enhancement of neurotrophic SirT1 in the brain has potential to modulate neuronal activity that may clinically provide an improvement in cognitive function and complement the current anti-Aβ antibody monotherapy. Our findings support further development of DDL-218 as a novel ApoE4-targeted therapeutic candidate for AD.

## Introduction

Sporadic Alzheimer’s disease (AD) currently affects an estimated 6.9 million Americans age 65 and older, and while the reported incidence of AD has decreased in recent years, increasing numbers of older people will ultimately lead to a surge of AD cases over the next two decades^1^. The hallmarks of AD brain tissue are amyloid plaques largely comprised of amyloid-β (Aβ), a product of amyloid precursor protein (APP) processing^2,3^, and neurofibrillary tangles, which form as a consequence of hyperphosphorylation of the protein tau^4^. More recent investigations have revealed, however, that AD is a multi-factorial disease, with a variety of factors increasing risk of both age of onset and rate of progression^5,6^.

While the familial forms of AD are associated with genetic variants in APP or the enzymes responsible for production of Aβ, including BACE1 and the executor of γ-secretase cleavage, presenilin 1^7–9^, the major risk factor for sporadic AD is expression of apolipoprotein E4 (ApoE4). Approximately 40–65% of AD patients possess either one or two ApoE4 alleles, with ApoE4 homozygosity now reconceptualized as a genetic form of AD^10^. Since confirmation of its implication in development of AD by Genome Wide Association Studies (GWAS)^11,12^, ApoE4 effects have been extensively studied^13–15^. While initially much attention was paid to ApoE4’s role in lipid metabolism that suggested it conferred greater risk for AD by impairing effective clearance of Aβ as compared to ApoE3 or E2^16^, knowledge concerning ApoE4’s range of pro-AD effects has expanded substantially in the last decade. The known pro-AD effects of ApoE4 include associations with increased Aβ production and seeding of pathology^17^, mitochondrial dysfunction, lysosomal leakage, increased site-specific tau phosphorylation, cytoskeletal disruption^18,19^, and regulation of gene transcription including that of the master metabolic regulator and neurotrophic factor, NAD-dependent histone and tau deacetylase sirtuin 1 (SirT1)^20^.

Based on our previous finding that ApoE4 affects expression of SirT1 by binding to the SirT1 gene promoter^20^ - acting as transcriptional ‘brake’ on SirT1 expression - and that lower SirT1 levels are implicated in AD^21^, we sought to identify a SirT1 enhancer that was efficacious in the presence of ApoE4 as a potential novel therapeutic for AD.

As we previously reported in Campagna et al. 2018^22^, our initial screening of a compound library produced the hit A03 that increased SirT1 while not affecting levels of neurotoxic sirtuin 2 (SirT2) in vitro, and both increased SirT1 in hippocampi of treated mice and improved working memory. Here, we describe our discovery of an analog of A03, DDL-214, and identification of the active enantiomer, DDL-218.

We further sought to elucidate the mechanism of action (MoA) of DDL-218 by use of affinity purification^23^ with a related analog and proteomics to reveal protein targets for compound interactions, chromatin immunoprecipitation (ChIP)^24^ to assess SirT1 gene promoter occupancy by RNA polymerase (RNAP) versus ApoE4, overexpression and knockdown studies to determine PRMT5 effects on SirT1 levels, OCTET analysis for PRMT5-ApoE4 interaction, and molecular dynamic simulation to model ApoE4 and variant interaction with the SirT1 promoter. To confirm efficacy in vivo, DDL-218 was tested in the ApoE4-TR:5XFAD mouse model. These studies reveal a novel mechanism by which DDL-218 treatment elicits de-repression, referred to here as ‘release of the brake’, of SirT1 expression by ApoE4 in the brain and improvement in memory.

## Materials and methods

### Discovery of DDL-218

DDL-218 was discovered during our ongoing medicinal chemistry and analoging efforts directed at increasing the potency, brain-permeability, and drug-like qualities of our initial SirT1 enhancer hit A03 (EC50 ~ 2.3 µM) described in Campagna et al. 2018^22^. Details of the medicinal chemistry efforts will be presented in a future manuscript. The synthesis scheme for DDL-214, the racemate from which enantiomer DDL-218 is derived, is described in Supplementary Methods and shown in Supplementary Figure S1.

The enantiomers of DDL-214 were separated by Supercritical Fluid Chromatography (SFC) as described in Supplementary Methods. The chromatograms for the separated enantiomers are shown in Supplementary Figure S2. Physiochemical properties of DDL-218, including kinetic solubility, brain tissue binding and microsomal stability were determined as described in Supplementary Methods and presented in Supplementary Table S2.

The assessment of optical rotation is presented in Supplementary Methods, and Supplementary Table S1 and Figure S3.

### Affinity purification, and PRMT over-expression in SH-SY5Y cells

To assist in identification of possible protein interactions of our SirT1 enhancers, affinity purification and STRING analysis-based proteomics was used, as described in Supplementary Methods. This analysis suggested interaction with a protein arginine methyltransferase (PRMT), which was identified by LC-MS/MS in high abundance in the enrichment analysis. To assess effects of various PRMTs 1, 4, 5, 7 and 8 on SirT1, N2a-E4 cells were transfected with each of these PRMTs as described in Supplementary Methods.

### In vitro testing of DDL-214 and enantiomers in N2a-E4 cells

Relative SirT1 mRNA and protein enhancement by racemate DDL-214 and enantiomers DDL-218 and − 219 was determined in murine neuroblastoma cells stably transfected with ApoE4 (N2a-E4)^25^. All test compounds in DMSO were prepared at 2x the final concentration in DMEM supplemented with 10% fetal bovine serum (FBS) and 1% pen-strep (PS) and 50 µL of each loaded into individual wells of a 96-well plate. Previously cultured N2a-E4 were then trypsinized, harvested, centrifuged, and counted; then 50 µL of cell suspension adjusted to 400 cells/µL was added to each well. The plate was then incubated at 37 ^o^C, 5% CO_2_ for 48 h. Cells were treated with DDL-214, DDL-218, or DDL-219 at 5 µM final then incubated for 24 h. After incubation, the cells were lysed with 50 µL of ice-cold lysis buffer (0.4% SDS, 3.5% Triton X-100, 0.02% sodium deoxycholate, 28 mM TRIS pH 8) complemented with 1X final Halt™ Protease and Phosphatase Inhibitor Cocktail (Thermo Fisher catalog # 78442), placed on ice for 10 min and at -80^o^C, followed by 3 freeze/thaw cycles.

An AlphaLISA comprising streptavidin donor beads (Revvity catalog # 6760002 S), biotinylated anti-SirT1 N-terminal antibody (monoclonal 19A7AB4, Abcam catalog # ab110304), and acceptor beads (Revvity catalog # 6772001) conjugated to the anti-SirT1 C-terminal antibody (monoclonal 1F3, Abcam catalog # ab104833) was used to determine SirT1 protein levels. For the AlphaLISA, a 384-Proxy plate (Revvity catalog # 6059480) was preloaded with 2 µL of the acceptor mixture (19A7AB4-biotin and 1F3-acceptor), then 2 µL of cell lysate was transferred to the proxy plate followed by incubation at room temperature for 1 h. Then, 2 µL of the donor bead mixture was added and incubated for an additional 30 min. The plates were read using an Envision plate reader.

### Testing in human Kelly neuronal cells

Human neuroblastoma ‘Kelly’ cells (genotype APOE3/APOE4) were plated in 96-well plates at 50K cells per well and were grown overnight in RPMI 1640 supplemented with 2 mM Glutamine, 10% FBS and 1% Pen-Strep. Cells were treated with 5 µM DDL-214, -218, or -219; or DMSO vehicle (*n* = 8 per compound). Twenty-four hours later, medium was removed, cells washed and lysed as described above for N2a-E4 cells.

The Kelly neuronal cells were obtained commercially while the N2a-E4 cells were gifted from the Bredesen lab.

### PRMT5 over-expression in N2a-E4 cells

For the assessment of the effects of PRMT5 over-expression on SirT1 levels in murine neuroblastoma cells stably transfected with apolipoprotein E4 (N2a-E4), 75K N2a-E4 cells/well were plated in a 24-well plate and cultured at 37 ^o^C with 5% CO_2_ in DMEM supplemented with FBS and G418 and grown overnight. Cells were then transfected with 0.75 µg of pCMV6-Entry vector (Origene # PS100001) or the human ORF clone for PRMT1 (Origene # RC224239), PRMT4 (Origene # RC217483), PRMT8 (Origene # RC205188), PRMT5 (Origene # RC203458), or PRMT7 (Origene # RC201672) using TurboFectin transfection reagent (Origene# TF81001) following manufacturer’s protocol. After 48 h, cells were washed with PBS followed by lysis using 70 µL MPER protein extraction reagent (Thermo Fisher Scientific # 78501) supplemented with protease phosphatase inhibitors (Thermo Fisher scientific # 78446). Cells were collected in pre-chilled 1.5 mL microcentrifuge tubes using a cell scraper and kept on ice for 15 min followed by centrifugation at 14,000 x g for 10 min at 4 ^o^C. The supernatant was collected and stored at -80^o^C until used. Protein concentration was determined by a BCA assay.

For immunoblotting, 15–20 µg of protein underwent electrophoresis using a 4–20% Tris-glycine gel (Thermo Fisher # XP04205BOX) followed by transfer to a PVDF membrane using BioRad Mini Trans-Blot Electrophoretic Transfer system (60 V for 2 h) (BioRad # 1703930). The membrane was probed overnight with the primary antibody against protein of interest (see Table 1) at 4 ^o^C, washed 3X with PBST, incubated with secondary antibodies from LiCOR, and scanned using the LiCOR Odyssey infrared scanner.

**Table 1:** Antibodies and dilutions used for immunoblots.

Antibody	Manufacturer	Catalog #	Dilution
PRMT1	Cell Signaling	2449 S	0.7361
PRMT4	Cell Signaling	4438 S	0.7361
PRMT8	Millipore	ABS517	3.5138
PRMT5	Cell Signaling	79,998	0.7361
PRMT7	Santa Cruz	sc-376,077	0.5625
Beta actin	Abcam	ab8226	3.5138

### PRMT5 SiRNA knockdown in N2a-E4 cells

To assess the effects of decreased PRMT5 expression (knockdown), N2a-E4 cells (*n* = 9 per condition) were cultured at 37^o^C in 5% CO_2_ using DMEM media supplemented with FBS and G418. For PRMT5 siRNA transfection, cells were plated at 50 K/ well in 24 well plate and grown overnight. Cells were transfected using 100 nM of PRMT5 siRNA (pool of 3 different siRNAs- A, B and C; Origene #SR418510) or 100 nM scrambled siRNA (provided with kit Origene #SR418510) for 48 h using siTran 2.0 reagent following manufacturer’s protocol (Origene # TT320001). After 48 h, cells were washed with PBS and lysed using 350 µL RLT guanidine thiocyanate buffer (Qiagen) for SirT1 and PRMT5 mRNA analysis or 65 µL MPER buffer supplemented with protease phosphatase inhibitors for protein analysis. For protein analysis, cells were collected in pre-chilled 1.5 mL microcentrifuge tubes using a cell scraper and kept on ice for 15 min followed by centrifugation at 14,000 x g for 10 min at 4 ^o^C. The supernatant was collected and kept at -80 ^o^C until assay. Protein concentration was determined by BCA assay. Immunoblots were performed as described above for PRMT5 overexpression, using the PRMT5 and beta actin antibodies listed in Table 1.

### Methylation analysis after ApoE4-PRMT5 incubation

Methylation of ApoE4 by PRMT5 and analysis were performed as described in Supplementary Methods.

### OCTET analysis

OCTET analysis of ApoE4-PRMT5 binding was performed to determine binding affinity, as described in Supplementary Methods.

### Molecular modeling of ApoE4, ApoE4(R251G) and ApoE4(R112-DA2) SirT1 CLEAR sequence binding energy and in vitro testing

A rare R251G mutation in E4 has been reported to neutralize the E4-dependent risk of AD^26^. To determine if this lowered risk is related to SirT1 expression, we assessed how the ApoE4(R251G) mutation affects its protein binding to the CLEAR (*C*oordinated *L*ysosomal *E*xpression and *R*egulation) transcription regulatory DNA site in the SirT1 gene promoter by in silico molecular modelling and dynamic simulations. We also performed molecular modeling using dimethylated ApoE4 (R112-*DA2*).

The effect of the R251G mutation on SirT1 mRNA expression was assessed by transfection of human SH-SY5Y neuroblastoma cell line (which has an E3/E3 genotype) with expression vectors for ApoE4 and ApoE4(R251G) (see Supplementary Methods).

### In vivo models and ethics statement

All procedures were performed in accordance with protocol approved by the UCLA Institutional Animal Care and Use Committee (IACUC) and guidelines of the NIH. All in vivo methods were performed in accordance with relevant guidelines and regulations in the approved protocol. Mouse strains used were C57BL/6J (Black 6, Jackson Laboratory; JAX stock #000564). All non-AD mice were 4 to 5 mo old at the time of the experiments. The AD model mice consisted of ApoE-TR:5xFAD mice which express APOE4 under the control of the endogenous mouse APOE promoter were bred to 5xFAD mice (Tg6799) which co-express five FAD mutations (APP K670N/ M671L + I716V + V717I and PS1 M146L + L286V) under the control of the neuron-specific mouse Thy-1 promoter, and backcrossed three times to ApoE-TR mice, resulting in mice homozygous for APOE4, and hemizygous for the 5XFAD transgenes, on a background strain 97% C57Bl/6J and 3% SJL; mice were then inbred between 5xFAD + and 5xFAD- resulting in littermates E4+/+:FAD + and E4+/+:FAD-. The AD model mice were 6-7.5 mo old for the in vivo studies. For all the in vivo testing both male and female mice were used.

### In vivo Pharmacokinetic analysis

C57BL/6J mice were administered DDL-214, -218, or -219 (*n* = 3 per compound) by oral gavage at a dose of 30 mg/kg in a volume of 5 mL/Kg based on mouse weight. One hour later, mice were euthanized by pentobarbital over-anesthesia and trans-cardial collection of blood for isolation of plasma by centrifugation at 10,000 x g for 10 min at 4 ^o^C. Brain tissue was collected for analysis, frozen on dry ice and stored at -80 ^o^C before analysis.

For LC/MS/MS analysis, tissue samples were homogenized in a bead beater using 5 volumes of ice-cold 80% acetonitrile (1/5; mg of tissue/ µL of 80% ACN). Solutions were clarified by centrifugation (16,000 x g, 5 min) and the supernatants were transferred to new tubes and lyophilized. Samples were reconstituted in 100 µL of 50/50/0.1 (water/acetonitrile/formic acid) before analysis via liquid chromatography-tandem mass spectrometry.

A 6460 triple quadrupole mass spectrometer (Agilent Technologies) coupled to a 1290 Infinity HPLC system (Agilent Technologies) with a Phenomenex analytical column (Kinetex 1.7 μm C18 100 Å 100 × 2.1 mm) was used for HPLC. The HPLC method utilized a mixture of solvent A (99.9/1 water/formic acid) and solvent B (99.9/1 acetonitrile/formic acid) and a gradient was used for the elution of the compounds (min/%B: 0/20, 3/20, 19/99, 20/99, 21/20, 30/20).

An internal standard (IS; Reserpine) was added to every sample to account for compound loss during sample processing. Standards were made in drug naïve brain and plasma with increasing amounts of DDL-214- (S1,S2: 0.1 pmol/ S3,S4: 1 pmol/ S5,S6: 10 pmol/ S7,S8: 100 pmol/ S9,S10: 1000 pmol). The standard curve was made by plotting the amount of each compound per standard vs. the ratio of measured chromatographic peak areas corresponding to DDL-214 (DDL-214 -/Reserpine). The trendline equation was then used to calculate the absolute concentrations of DDL-214/-218/-219 in brain tissue and plasma.

### In vivo efficacy testing in ApoE4-TR:5xFAD mice

The active (-) enantiomer, DDL-218, underwent efficacy testing in ApoE4-TR:5xFAD mice^27^ that were 6-7.5 months old at the start of treatment. ApoE4-TR:5xFAD male and female mice received either 20 mg/Kg DDL-218 BID (~ 8- to10-hour interval; total daily dose of 40 mkd) on weekdays and a single 40 mg/Kg dose on weekends (*n* = 12) or vehicle-only (*n* = 11); 8 C57BL/6 mice received vehicle-only as behavioral controls. Dosing was by the oral route and by oral feeding using a pipette as previously described^28^, not gavage, as it is safer for repeated oral dosing than gavage. The dose of 20 mg/kg BID (40 mkd) was selected based on findings from PK analysis and was the highest feasible in the formulation used for oral feeding (compound is first solubilized in water, then mixed strawberry syrup diluted in water, for a final vehicle that is 25% syrup and 75% water). The final concentration of DDL-218 was chosen to deliver 20 mg/Kg in a volume of 10–20 µL (adjusted to the weight of each mouse). Mice were treated for 56 days (8 weeks); in week 7, mice underwent Barnes maze testing^29^ as described below.

On Day 56, mice were euthanized 2 h after the a.m. dose by pentobarbital over-anesthesia and cardiac puncture for the collection of blood in EDTA tubes on ice for the isolation of plasma by centrifugation at 3,000  x g for 10 min at 4 ^o^C. Mice were perfused with saline at 5 ml/min. Whole brains were removed and halved down the mid-line and combined hippocampal/entorhinal cortex (Hip/ECx) and frontal cortex (FrCx) for biochemical and proteomics analyses.

### Barnes maze testing

Mice underwent Barnes Maze^29^ testing at week 7 of treatment. The Barnes Maze is a white circular platform that is 36 inches in diameter with 20 holes around the periphery (San Diego Instruments). Testing comprised Day 1 habituation to the maze wherein mice were placed at the center of the maze inside the start chamber. After 10 s, the start chamber was removed and mice were allowed to explore the maze for total 5 min: after 3 min of exploration, the mouse was guided to the escape hole. Habituation was followed by 3 days of training (‘acquisition’) wherein the mouse learns to locate the escape hole. A mouse is placed at the center of the maze inside the start chamber and after 10 s, is released and allowed to search for the escape hole. Mice navigate by visual cues on walls surrounding the maze on which large graphic signs are displayed that are different for each of the 4 walls. Three trials a day were performed by each mouse. After entry of the escape hole, the mouse was allowed to stay until the end of the trial or for 1 min. If the mouse did not find the escape hole within 3 min, it was guided to the hole and allowed to stay for 1 min before being returned to home cage.

For the probe test of memory, 48 h after the last training, the escape hole is closed and the escape box removed. Mice are placed at the center of the maze inside the start chamber. After 10 s, the start chamber is removed, and mice are allowed to explore the maze for 90 s. At the end of the trial, the mice are returned to the home cage. Some mice were immobile during the probe and were excluded from group data analysis. Analysis of latency over the course of training trials was performed using ANY-Maze Software (Wood Dale, IL) and probe data was calculated manually as the software excludes mice that do reach the escape hole within 90 s; in manual scoring, they are assigned a 90 s latency.

### Global proteomics on brain tissue

Hippocampal brain tissue from both DDL-218- and vehicle-treated mice underwent global proteomics analysis, as described in Supplementary Methods.

### Gene expression mRNA assays

Total RNA was isolated from cells or brain tissue (Qiagen RNEasy kit). Total RNA (0.5–1.0 µg) mRNA was then converted to cDNA (SuperScript™ III First-Strand Synthesis SuperMix kit; Thermo Fisher), and gene mRNA levels were measured using a gene-specific qRT-PCR Taqman assays (Thermo Fisher). Gene mRNA levels were normalized to GAPDH mRNA levels measured either in the same cDNA sample or in the same qPCR reaction using multiplexing of gene-of-interest/VIC and GAPDH/FAM, normalized by the delta-delta-Ct method.

### Statistical methods

For comparison of two groups, a two-tailed unpaired student’s t-test was used. For groups of 3 or more one-way ANOVA was used, with Tukey’s post-hoc comparison of groups, where*p ≤ .05, **p < .01, ***p < .001 and ****p < .0001. The statistical method we use assumes normal distribution and will be verified in future studies using increased n numbers.

## Results

### DDL-218, the active (-) enantiomer of DDL-214, increases SirT1, NFYb, and PRMT5

**Fig. 1:**
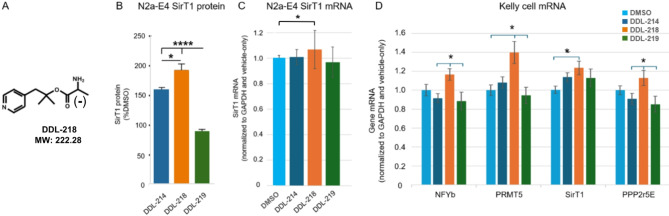
DDL-214 racemate and enantiomer effects on target mRNA and proteins. (**A**) The structure and molecular weight of DDL-218. Levels of SirT1 (**B**) protein (*n* = 6) and (**C**) mRNA (*n* = 5 for DDL-214 and 218; *n* = 6 for DDL-219) in murine N2a-E4 cells after treatment with DDL-214 (racemate), or DDL-218(-) enantiomer or DDL-219(+) enantiomer at 5 µM for 24 h. (**D**) Levels of NFYb, PRMT5, SirT1, and PPP2r5e mRNA in human Kelly neuroblastoma cells treated with DDL-214, DDL-218, or DDL-219 (*n* = 8). Data normalized to DMSO control. Data graphed as the mean and SEM. Statistical analysis performed for (**B**) and (**D**) using one-way ANOVA and Tukey’s post-hoc comparisons, where*p ≤ .05 and ****p < .000.1 An unpaired student’s t-test was performed for the comparison in (**C**) (one-way ANOVA was not significant). mRNA data are normalized to GAPDH mRNA in the same samples and to levels in DMSO (veh)-treated cells.

We utilized iterative medicinal chemistry to identify an analog, DDL-214 (Fig. 1A), of our original SirT1-enhancing hit A03^22^ that increased SirT1 protein levels in ApoE4-expressing N2a (N2a-E4) murine neuroblastoma cells (Fig. 1B). The mean for SirT1 mRNA in human ApoE3/4-expressing Kelly neuroblastoma cells^30^ treated with DDL-214 was higher than DMSO control, but the difference was not significant (Fig. 1D).

The synthesis scheme for DDL-214 is described in the Supplementary Methods (Fig. S1). The enantiomers of racemate DDL-214 were separated by supercritical fluid chromatography (SFC), and optical rotation of the enantiomers DDL-218 and 219 (Supplementary Methods; Figures S2 and S3A, and Table S1) and effects on SirT1 levels in both N2a-E4 and Kelly cells assessed. The active enantiomer that elicited the greatest increase in SirT1 protein in N2a-E4 cells at 5 µM after 24-hour exposure (Fig. 1B) and in SirT1 mRNA in N2a-E4 (Fig. 1C) and Kelly cells (as compared to DMSO control) (Fig. 1D) was found to be the (-) enantiomer, DDL-218. The racemate DDL-214 in N2a-E4 cells gave similar increase of SirT1 protein levels at 5 and 50 μm when compared to the hit A03^22^ (Supplementary Fig S3B).

To assist in identifying proteins with which the SirT1 enhancer may interact, affinity purification was performed using DDL-220 an alkyne-containing SirT1 enhancer analog that was clicked to affinity column agarose magnetic beads (Supplementary Methods). This analysis revealed that a protein arginine methyltransferase (PRMT) interacts with DDL-220 (Fig. S4), a compound in this series, and thus may have a role in DDL-214/-218 enhancement of SirT1. The class-I PRMTs (1, 4, 8), the class-II PRMT5, and class-III PRMT 7 ^31^ were tested by overexpression in N2a-E4, wherein PRMT5 (but not PRMTs 1, 4, 7 and 8) produced a significant increase in SirT1 (Supplementary Fig. S5A–D). Subsequently, it was confirmed that DDL-218 significantly increases PRMT5 expression (Fig. 1D).

Based on the report of Zhang et al. 2014^32^, who demonstrated the transcription factors NFYa and NFYb control and upregulate PRMT5 expression in prostate cancer cells, we also determined levels of these transcription factors in DDL-218-treated human neuronal Kelly cells, which showed DDL-218 treatment only significantly increases NFYb mRNA compared to DMSO-treated cells (Fig. 1D).

Because levels of PPP2r5e mRNA have also been described as affected by ApoE4^20^, the effects of treatment on PPP2r5e mRNA in Kelly cells was also determined, and it was also found to be significantly increased by DDL-218 compared to DMSO-treated cells (Fig. 1D). Further, the DDL-218 induced increases in NFYb, PRMT5, PPP2r5e and SirT1 mRNA levels were significantly greater than DMSO treated cells as compared to the antipode enantiomer DDL-219, in Kelly cells.

Correlations between each of the gene mRNAs are shown in Supplementary Figure S6 and were significant for PRMT5 vs. NFYb are *R* = .7484, R^2^ = 0.5601, and p 0.000026; for SirT1 vs. PRMT5 are *R* = .6106, R^2^ = 0.3728, and *p* = .00153; and for PPP2r5e vs. SirT1 are *R* = .552, R^2^ 0.2998, and *p* = .005164.

ChIP analysis of SirT1 promoter occupancy by RNA polymerase (RNAP) and ApoE4 (see Supplementary Methods) in Kelly cells showed that RNAP occupancy was increased by DDL-218 and ApoE4 binding was lower, but the effect was not significant (Supplementary Fig. S7A).

### PRMT5 overexpression increases, and knockdown decreases, SirT1 levels in N2a-E4 cells

**Fig. 2:**
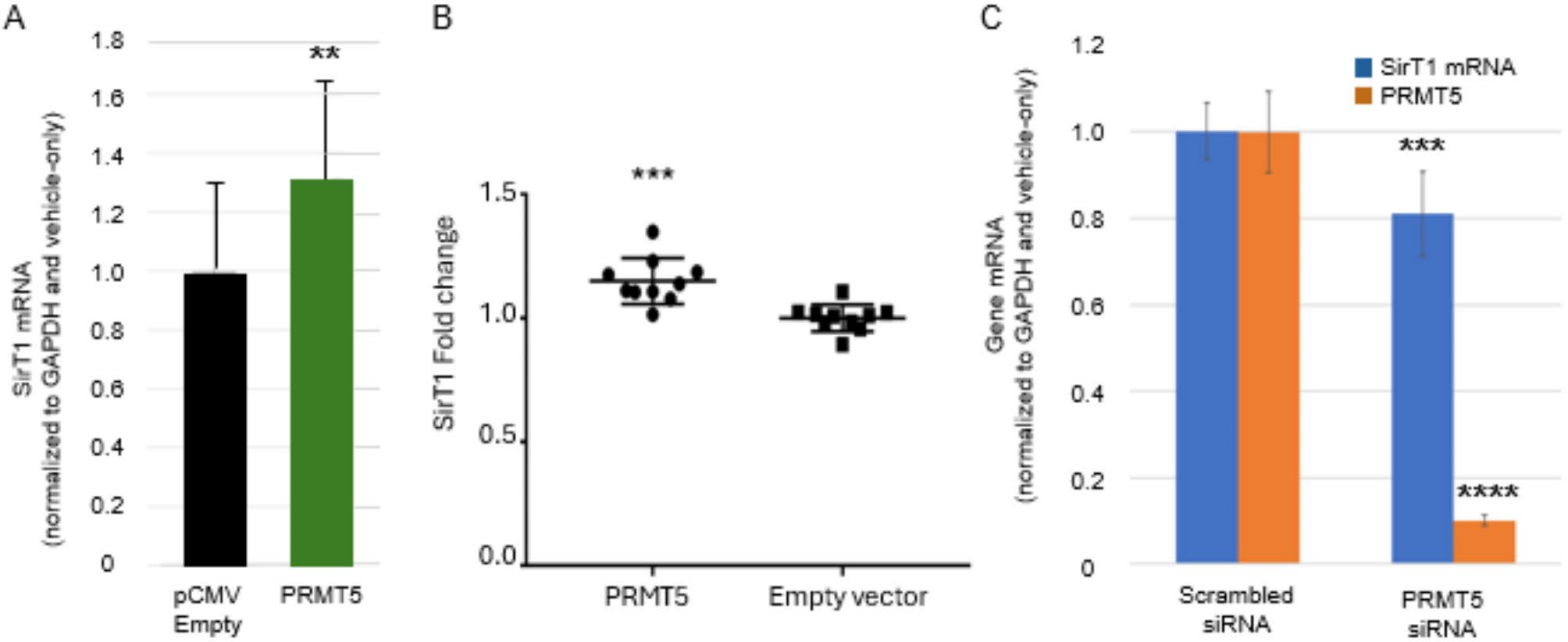
PRMT5 overexpression increases,* and knockdown decreases*,* SirT1 expression.* (**A**) SirT1 mRNA levels after transfection of N2a-E4 cells with either empty pCMV vector or a PRMT5-expressing vector (over-expression) (*n* = 16). (**B**) Fold change in SirT1 protein level after PRMT5 vs. empty vector transfection. (**C**) The levels of SirT1 and PRMT5 mRNA normalized to GAPDH, and GAPDH mRNA levels (not normalized) after transfection of N2a-E4 with vehicle (untreated), scrambled siRNA or PRMT5 siRNA (*n* = 9). Statistical analysis performed using an unpaired, two-tailed student’s t-test where **p < .01 and ***p < .001. Comparison in (B) is between scrambled siRNA and PRMT5 siRNA.

To elucidate the association of PRMT5 expression and SirT1 enhancement, N2a-E4 cells were transiently transfected with a pCMV PRMT5-expressing construct. Forty-eight (48) hours after transfection, PRMT5 protein levels were significantly higher in cells treated with the PRMT5-expressing construct than with the empty pCMV vector (Supplementary Fig. S7B), and this PRMT5 increase was associated with a significant, approximately 30% increase in SirT1 mRNA (Fig. 2A). The fold change in SirT1 protein was also significantly different between PRMT5 and empty vector-transfected cells (Fig. 2B).

ChIP analysis was also performed to ascertain effects of PRMT5 overexpression, which was found, similarly to DDL-218, to increase RNAP and decrease ApoE4 occupancy of the SirT1 promoter (Supplementary Fig. S7C).

Conversely, an approximately 10-fold knockdown of PRMT5 expression by transfection of N2a-E4 cells with PRMT5 siRNA was associated with a 19% decrease in SirT1 mRNA (Fig. 2C).

### PRMT5 shows moderate binding to ApoE4 in OCTET analysis

Based on the findings described above, our hypothesis is that PRMT5 interacts with ApoE4 and may compete with the SirT1 promoter for binding of ApoE4, acting to reverse the repression of SirT1 expression by ApoE4. To provide support for this hypothesis, a kinetics assay was performed on the Octet platform using biotinylated ApoE4 and various concentrations of a recombinant PRMT5/MEP50 complex. The steady state graph derived from this analysis revealed PRMT5/MEP50 complex to have moderate binding affinity for ApoE4, with an estimated *K*_D_ of 970 nM (Supplementary Fig. S8).

### Molecular modeling of ApoE4wt ApoE4(R112-DA2) and ApoE4(R251G)-SirT1 promoter interaction and in vitro testing of ApoE4(R251G)

In our in silico molecular modeling, we used ApoE4 structures modeled from ApoE3 Protein Data Bank (PDB): 2L7B) by replacing Cys 112 with Arg^33^ for ApoE4, with both C112R and an Arg 251 to Gly substitution for ApoE4(R251G), and with asymmetrically dimethylated Cys112Arg for ApoE4(R112-*DA2*). Binding of each of these models to the SirT1 CLEAR DNA regulatory site was simulated. Molecular dynamic simulation predicted weaker and less stable interactions of ApoE4(R112-*DA2*) and ApoE4(R251G) with CLEAR as compared to ApoE4wt during the 100-ns simulation [link to Supplementary Video 1; Supplementary Video 2]; whereas ApoE4 forms a stable complex with the CLEAR DNA motif throughout the entire 100-ns simulation [link to Supplementary Video 3]. Modeling also predicted that on average, ApoE4 formed twice as many hydrogen bonds with the CLEAR DNA sequence as ApoE4(R112-*DA2*) or ApoE4(R251G). As shown in Supplementary Figure S9, for ApoE4 ΔG = -65.27 ± 11.54 kcal/mol, ApoE4(R112R-*DA2*) ΔG = -42.38 ± 16.96 kcal/mol and for ApoE4(R251G) ΔG = -46.74 ± 18.32 kcal/mol.

In vitro comparison of ApoE4 and ApoE4(R251G) supported the modeling predictions. In apolipoprotein E3-expressing (E3/3) SH-SY5Y human neuroblastoma cells^34–36^ electroporated with ApoE4, SirT1 mRNA expression was significantly reduced by 28% compared to pCDNA3-electroporated control cells, whereas electroporation with an ApoE4(R251G)-expression vector reversed the decrease of SirT1 mRNA levels, which was similar to the levels in control pCDNA3-electroporated control cells (Supplementary Fig. S10).

### Methylation analysis after ApoE4-PRMT5 incubation

Because methylation of the ApoE4 arginine at position 112 was predicted to lower interaction with the CLEAR sequence, as observed in molecular dynamics simulations with ApoE4(R112-*DA2*), we determined if the mechanism by which PRMT5 – with a known role in methylation – decreased ApoE4 interaction with the SirT1 promoter was via methylation of ApoE4, but no methylation was detected (Supplementary Fig. S11).

#### DDL-218 in vivo

##### Pharmacokinetics of DDL-214, DDL-218, and DDL-219

In a time course pharmacokinetics (PK) study of DDL-214 racemate, the brain C_max_ after oral gavage delivery of 10 mg/Kg to a wildtype mouse was 1270 ng/g (5.7µM) in brain at one hour; the C_max_ for oral 30 mg/Kg was ~ 9400 ng/g (42.3 µM) (Supplementary Fig. S12A). To ascertain if the racemate and enantiomers were brain-penetrant, brain levels were determined at a single time point of one hour and an oral gavage dose of 30 mg/Kg (*n* = 1). In that experiment, brain levels for DDL-214, DDL-218, and DDl-219 were ~ 3000 ng/g (13.5 µM), ~ 8400 ng/g (37.8 µM), and ~ 6500 ng/g (29.2 µM) in brain, respectively (Supplementary Fig. S12B). No notable changes in mice behavior that would be indicative of drug-induced toxicity were observed.

### In vivo study of DDL-218 in ApoE4-expressing mice

**Fig. 3:**
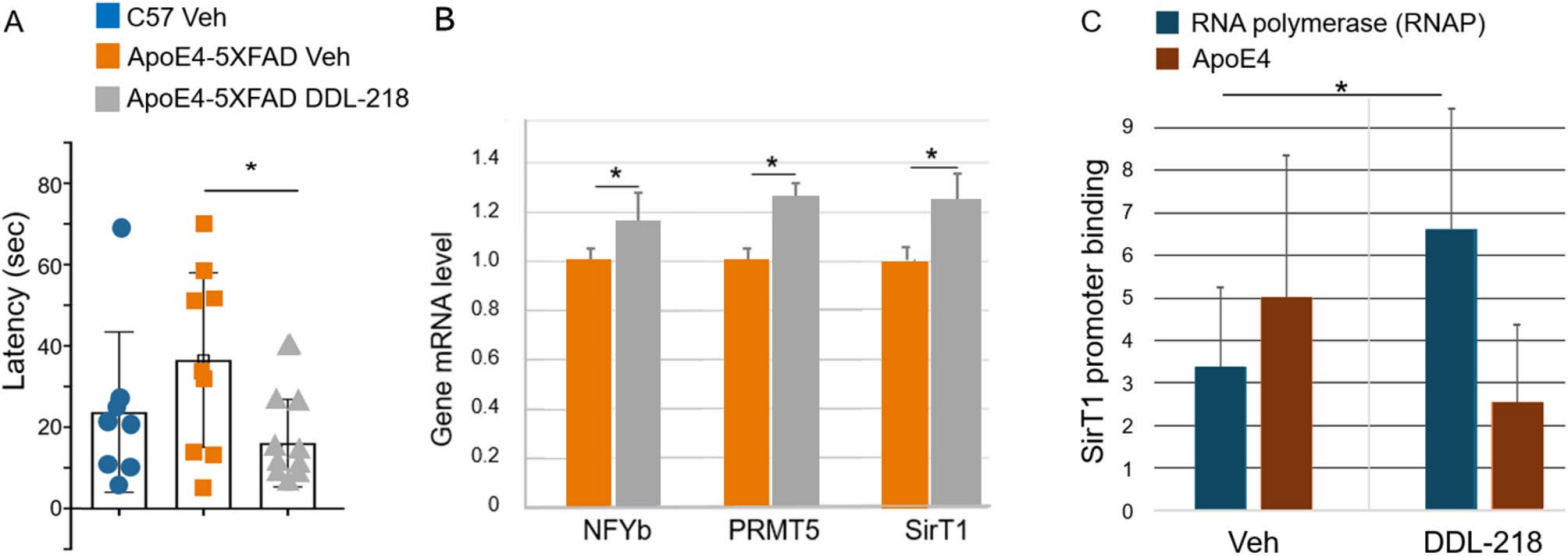
Barnes maze latency, gene mRNA levels, and SirT1 promoter occupancy. (**A**) Latency in seconds (sec) for mice to reach escape hole in the 48-hour probe in the Barnes maze. Mice who were immobile are not represented. (**B**) Gene mRNA levels for NFYb, PRMT5, and SirT1 in hippocampus of study mice, the color code in (**A**) also applies to (**B**). (**C**) Occupancy of the SirT1 promoter by RNAP or ApoE4 in brain tissue of ApoE4-TR:5xFAD mice treated with either vehicle-only (Veh) or DDL-218. Data graphed as mean and SEM. Statistical analysis performed using one-way ANOVA with Tukey’s post-hoc comparison of groups in (**A**) and by unpaired two-tailed student’s t-test for (**B**) and (**C**), where *p ≤ .05, for comparison of ApoE4-5XFAD Veh vs. DDL-218 mice in (**A**) and between RNAP promoter binding in Veh vs. DDL-218 in (**C**).

Overall, non-transgenic C57B6 mice had shorter latency in finding the escape hole in the Barnes maze in both training and the probes (Supplementary Fig. S13A). Latency for female and male mice is shown in Supplementary Figure S13B and C. In the 48-hour post-training probe, as compared to ApoE4-TR:5xFAD mice that received vehicle only, ApoE4-TR:5xFAD mice treated with 20 mg/Kg DDL-218 BID for 56 days displayed a decrease in latency to enter the escape hole in the Barnes Maze (Fig. 3A). Mice that did not move in the probe (stayed in the center) were removed from analysis (1 Veh mouse; 2 DDL-218) mice.

As compared to vehicle-treated ApoE4-TR:5xFAD mice, hippocampal NFYb, PRMT5, and SirT1 mRNA was significantly increased in DDL-218-treated ApoE4-TR:5xFAD mice (Fig. 3B). Levels of mRNA for these readouts for females and males are shown in Supplementary Figure S13D-F. The mean for SirT1 protein levels in hippocampi showed a similar increase of ~ 20%, but the difference was not statistically significant due to variability amongst mice, as shown for all mice, females, and males in Supplementary Fig. 13G.

ChIP analysis was also performed with brain tissue of mice (pools of two mice each for sample, matched for sex and SirT1 mRNA levels) treated with vehicle or DDL-218. As compared to Veh-treated ApoE4-TR:5xFAD mice, RNAP binding to the SirT1 promoter was significantly increased in DDL-218-treated mice and ApoE4 binding was lower, but the effect was not significant (Fig. 3C).

Unbiased global proteomics analysis of hippocampal brain tissue from DDL-218- and vehicle-treated mice in the in vivo study revealed several differentially expressed proteins (Supplementary Table S4 and Figure S14), including upregulated proteins PTprn2, Nubpl, Tbc1d17, Scfd2, and Shisa6.

## Discussion

Our findings described herein confirm a mechanism by which ApoE4, the major genetic risk factor for sporadic AD, may decrease SirT1 and elucidate a novel mechanism for DDL-218, the active enantiomer of DDL-214, reversal of ApoE4 repression of SirT1 expression through binding to the SirT1 promoter and acting as a transcriptional ‘brake’ on SirT1 gene expression. We show that this reversal by DDL-218 is through enhancement of the transcription factor NFYb and increase in PRMT5. Our studies also confirm, by both PRMT5 over-expression and knockdown, that SirT1 levels are positively associated with PRMT5 levels in N2a-E4 cells. Specifically, PRMT5 over-expression displaced ApoE4 and increased RNA polymerase occupancy of the SirT1 promoter. We observe a similar displacement of ApoE4 and increase in RNA polymerase on treatment of human neuronal Kelly cells with DDL-218. Our hypothesis for this displacement effect is that PRMT5 interacts with ApoE4 and competes with the SirT1 promoter for binding with ApoE4. This is supported by our OCTET kinetics analysis of PRMT5/MEP50 binding to ApoE4, with a moderate binding K_D_. This concurs with the report by Dansu et al. 2022 ^37^ showing ApoE4 to be a binding partner for PRMT5.

The association between NFYb, PRMT5 and SirT1 mRNA levels is further supported by the observation that the greatest increase in SirT1 was associated with the greatest increases in NFYb and PRMT5 in Kelly cells treated with DDL-214 or DDl-218. This significant correlation provides evidence that these compounds modulate a transcriptional regulation pathway leading from NFYb through PRMT5 to SirT1. In addition, mRNA levels of SirT1 and PPP2r5e were significantly correlated, consistent with their reported coregulation by ApoE4 ^20,38^ and t﻿heir significant correlation (Pearson correlation 0.914) in bioinformatic analysis of gene co-expression of 2300 human brain RNAseq samples (see Supplementary Figure S6).

Our in silico modeling and molecular dynamic simulation studies revealed ApoE4 has relatively high SirT1 promoter-binding free energy, followed by ApoE4(R112-*DA2*) and ApoE4(R251G). The reduced affinity of ApoE4(R112-*DA2*) makes it similar to ApoE3 binding compared to ApoE4 binding for the CLEAR DNA sequence and is consistent with other in silico studies^39,40^ and biophysical reports^20,41^ that found only ApoE4wt has the potential to bind the CLEAR motif with specificity and relatively high affinity.

Our modeling also suggests ApoE4(R251G) binds the SirT1 promoter with lower affinity than ApoE4, and therefore can reverse inhibition of SirT1 expression. This alteration in binding affinity is likely due to conformational changes in ApoE4(R251G) that affect its function^42,43^, including oligomerization^26,44^, the C-terminal alpha-helix structure domain interaction essential for E4 phenotypes^45^, and/or the N-terminal fragment (1-151) ‘active’ DNA binding domain of apoE4 ^46^.

Consistent with this modeling results, our in vitro data indicate SirT1 mRNA levels are restored by transfection with ApoE4(R251G) plasmid compared to ApoE4 in SH-SY5Y; this is the first reported biochemical phenotype of E4(R251G) to our knowledge. This effect may contribute to the protection conferred by the mutation, which is associated with an approximate 50% reduction in AD risk^26^.

**Fig. 4:**
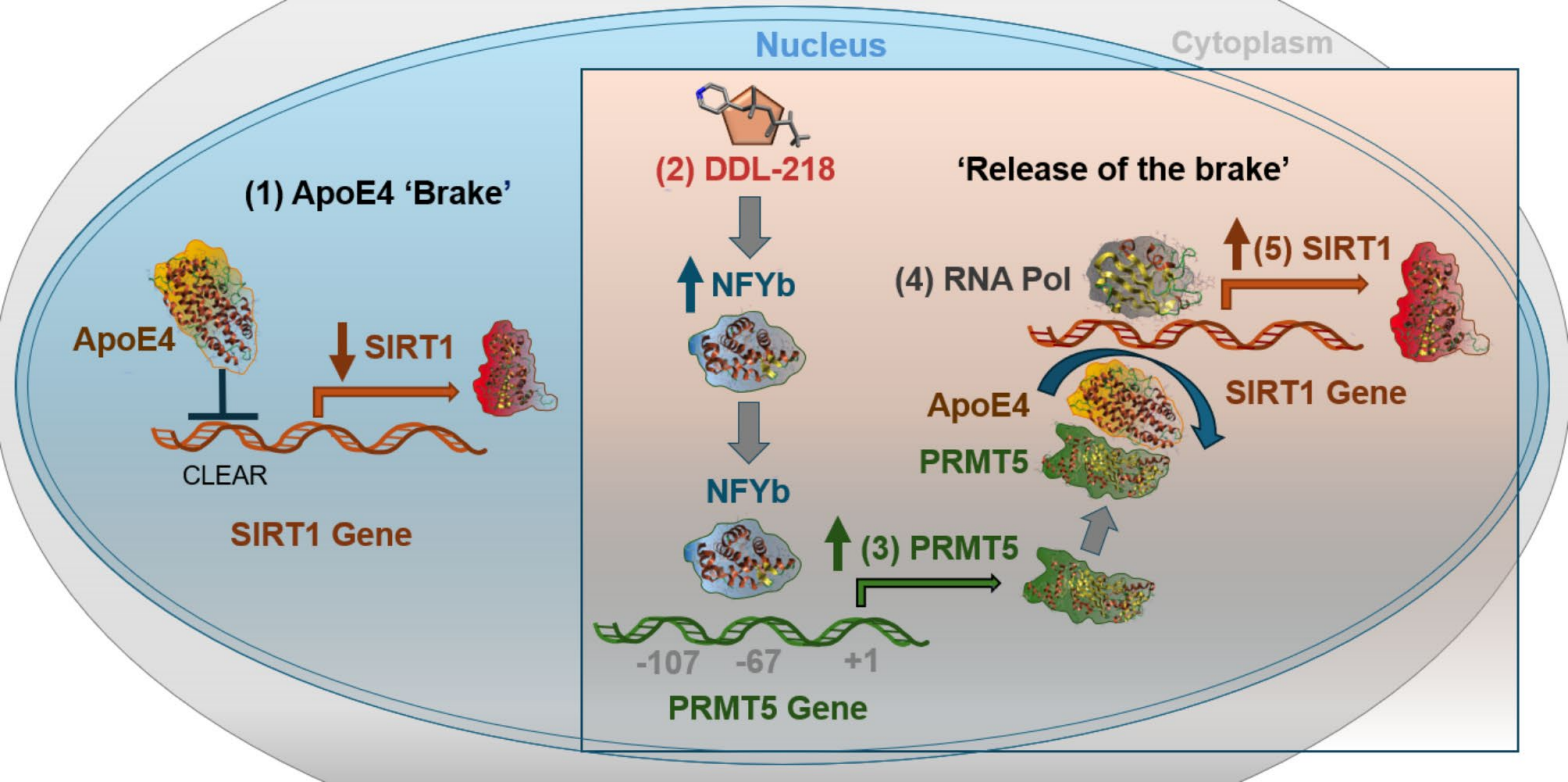
Proposed mechanism of action for SirT1 enhancement by DDL-218. (1) ApoE4 binds the SIRT1 promoter CLEAR sequence, acting as a transcriptional brake (repressor) and reducing SirT1 expression. (2) Small molecule DDL-218 increases expression of transcription factor NFYb, which then leads to (3) increased PRMT5 expression. (4) PRMT5 then binds ApoE4, decreasing ApoE4 occupancy of the SirT1 promoter, results in de-repression of SirT1 promoter or ‘release of the brake’. (5) This allows for increased RNA polymerase binding to the SIRT1 promoter, and increased SirT1 expression. The 3D protein images of ApoE4, NFYb, PRMT5 and RNA Pol were generated using Cresset software from PDB: 2L7B (ApoE4), 8QU3 (NFYb), 4GQB (PRMT5), 4A45 (RNA Pol).

We also demonstrate the in vitro and in vivo efficacy of our brain-permeable SirT1 enhancer lead candidate DDL-218, which significantly increased SirT1 mRNA in both N2a-E4 and human Kelly neuroblastoma cells, and in hippocampus/entorhinal cortex of ApoE4-TR:5xFAD mice after 56-day treatment at 40 mg/kg/day. DDL-218 also enhanced the transcription factor NFYb and PRMT5 mRNA in vivo. As further confirmation of our proposed mechanism (Fig. 4), RNA polymerase binding to the SirT1 promoter was greater with DDL-218 compared to vehicle-only treatment in Kelly cells and in brain tissue of ApoE4-TR:5xFAD mice. DDL-218 thus acts to reverse ApoE4 brake and transcriptional repression via increasing PRMT5, an action also supported by its increasing PPP2r5e levels, a gene which is also known to be repressed by ApoE4 ^20^.

NF-Y transcription factor (TF) that is modulated by DDL-218, is comprised of 3 subunits (a, b, and c), regulates ~ 25% of eukaryotic gene promoters, especially those controlling metabolism, cell-cycle, and stress response, acting as a “pioneer” TF^47^. NF-Yb is involved in upregulation of PRMT5 that is predominantly expressed in neuronal cells in human brain tissue^48^. How DDL-218 increases the mRNA levels of NF-Yb will be addressed in future studies seeking protein targets of DDL-218.

DDL-218 represents a novel small molecule transcription factor modulator with good drug-like properties, including good kinetic water solubility (found to be 64.4 µM), and a hERG (human Ether-a-go-go-Related Gene) EC50 > 10 µM, for targeting ApoE4 suppression of SirT1 levels. The 56-day oral treatment of ApoE4-expressing AD model mice with DDL-218 that demonstrated target engagement by enhancement of SirT1 mRNA and improvement in memory was not associated with any observable adverse effects, suggesting this compound could be a promising lead candidate for AD. We realize the limited ability of animal models to predict efficacy for complex neurodegenerative diseases such as AD^47^, however, the preclinical data showing enhancement of the neurotrophic SirT1 and improvement in memory suggests that DDL-218 has the potential to address pathology-related alterations in synaptic plasticity, neuronal function and pathways, leading to improvement in cognitive function, which could complement the lack of memory improvement achieved by Aβ-targeted antibodies. SirT1 enhancement may also protect neurons from other stressors not related to Aβ accumulation.

DDL-218 treatment of AD model mice resulted in differential expression of several proteins in the hippocampus (Table S4, Figure S12A). One upregulated protein, PTprn2 (protein tyrosine phosphatase receptor type N polypeptide 2), has been reported to be reduced in AD^48,49^. Gene ontology analysis (Supplementary Figure S12B) for PTprn2 indicates a role for PTprn2 in neurotransmitter release and synaptic plasticity and lower levels of PTprn2 may be associated with behavioral and learning impairments in mice^50^. In addition, other key genes identified as being upregulated were found to be involved in either mitochondrial function (Nubpl and Tbc1d17) or synaptic plasticity (Scfd2, and Shisa6) – functions that are implicated in AD pathogenesis (Supplementary Figure S12C). Thus, DDL-218 treatment may modulate both ApoE4 repression of SirT1 along with other proteins and pathways involved in neuronal function and memory.

In future preclinical studies, we will establish DDL-218 selectivity, and safety profile, and will test the compound in other ApoE4-expressing in vivo models. Further preclinical, analysis of AD-related biomarkers and IND-enabling studies would be needed for development of this potential new ApoE4-targeted, SirT1-enhancing therapeutic candidate for AD.

## Electronic supplementary material

Below is the link to the electronic supplementary material.

Supplementary Material 1.

Supplementary Video 1.

Supplementary Video 2.

Supplementary Video 3.

## Data Availability

All data generated or analyzed during this study are included in this published article and its Supplementary Materials/Information file. The mass spectrometry proteomics data have been deposited in the ProteomeXchange Consortium via the PRIDE repository with the identifier PXD060379 and DOI: 10.6019/PXD060379. Data are available via ProteomeXchange with identifier PXD060379 and Token: VZTkmqZDWowX.
