# Supplementary material for: Discovery of an ApoE4-targeted small-molecule SirT1 enhancer for the treatment of Alzheimer’s disease
Source: Sci Rep. 2025 Apr 23;15:14028. doi: 10.1038/s41598-025-96131-2 (PMC12019328; doi:10.1038/s41598-025-96131-2)
Supplement: Supplementary file 1 — Supplementary Material 1. [file 41598_2025_96131_MOESM1_ESM.pdf]

Supplementary Information for

Discovery of an ApoE4-Targeted Small-Molecule SirT1 Enhancer for the Treatment of Alzheimer's Disease

Jesus Campagna[1ξ], Sujyoti Chandra[1ξ], Bruce Teter[1], Whitaker Cohn[1], Johnny Pham[2], Young-Sug Kim[2], Barbara Jagodzinska[1], Kanagasabai Vadivel[1], Parvez Alam[1], Tina Bilousova[1], Malaney Young[1], Chris Elias[1], Juan Mariucci[1], Ilinca Flacau[1], Ainsley Jackman[1], Samar Padder[1], Dongwook Wi[1], Chunni Zhu[1], Patricia Spilman[1], Michael E Jung[2], Dale E Bredesen[1], Varghese John[1]*

[1]The Drug Discovery Lab, Department of Neurology, David Geffen School of Medicine, 710 Westwood Plaza, University of California Los Angeles, Los Angeles, CA 90095, USA

[2] Department of Molecular and Medical Pharmacology, 650 Charles E. Young Drive, University of California Los Angeles, Los Angeles, CA 90095, USA

[ξ] These two authors contributed equally

***Correspondence**: email vjohn@mednet.ucla.edu; phone 310-206-4345

DDL-214 Synthetic Scheme ……………………………..……………………………………….…...…3

Figure S1. *Synthetic approach for DDL-214*……………………………………………………..….3

SFC separation of DDL-214 enantiomers………………………………………………….…..…3

Figure S2. *HPLC-UV chromatograms for DDL-214 enantiomers*……………………..……………..4

Table S1. Optical rotation of enantiomers; SirT1 levels for DDL-214 & A03……………..….5-6

Figure S3A *Optical rotation of DDL-218 and -219* ………………………………….………………5

Figure S3B *SirT1 levels in N2a-E4 cells with DDL-214 and hit A03*…………………..……………6

Table S2. DDL-218 Physiochemical properties……………..…………………………..…..…….7

Affinity purification and STRING analysis..7

Figure S4. *Synthesis of column, affinity purification, and analysis*……………………………..8

Effects of PRMT over-expression on SirT1..8

Figure S5. *PRMT over-expression and SirT1 protein levels*…………………………………………10

Correlations between gene mRNAs levels in treated Kelly cells……………………………...11

Figure S6. *Correlations of gene mRNAs in Kelly cells*………………………………………………11

SirT1 promoter binding in transfected or treated N2a-E4 cells (ChIP) 11

Figure S7. *SirT1 promoter binding by RNA polymerase and ApoE4*..12

OCTET assay 13

Figure S8. *OCTET analysis of PRMT5/MEP:ApoE4 binding*……………………………………....13

***In silico* molecular modeling** 14

Figure S9. *Molecular modeling of ApoE4 and SirT1 CLEAR sequence*………….……………..….15

In vitro testing of ApoE4(R251G) in SH-SY5Y cells……………………...……………………15

Figure S10. *SirT1 mRNA levels in electroporated SH-SY5Y*……………………..…………..……16

Methylation analysis of ApoE4 by PRMT5…….……………………………………………...……16

Figure S11. *ApoE4 methylation analysis*…………………………………………………..……...…17

Figure S12. *Pharmacokinetics of DDL-214, -218, and -219 in vivo*………………………………..17

Figure S13. *Barnes Maze latency by sex, SirT1 protein in brain*…………………..………………18

Global Proteomics…………………………………………………………………………………..…19

Table S3. *Global Proteomics*……………..………………..……………………………………..…….20

Figure S14. *Proteomics analysis in brain with DDL-218 treatment*……………………………….…21

NMR, MS, and HPLC Analysis Figure S13 &14 Data.……..............…………………………..22

Supplementary References……………………………………………………….……………….25

DDL-214 Synthetic scheme

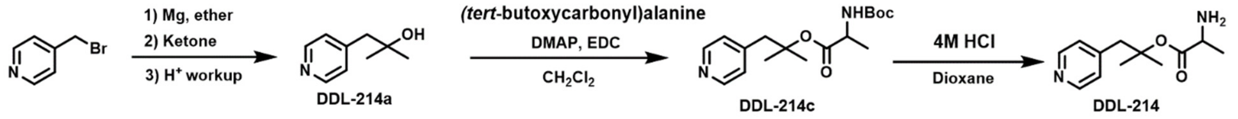

Figure S1. Synthetic approach for DDL-214.

DDL-214a (2-methyl-1-(pyridin-4-yl) propan-2-ol):

A dry 3-neck reaction flask equipped with a stir bar and reflux condenser was charged with magnesium turnings (22.6 mmol) and anhydrous Et_2O (20 mL). A solution of the 4-(bromomethyl) pyridine (15.1 mmol) in Et_2O (5 mL) was added dropwise from a pressure-equalizing additional funnel under argon for 10 min. The reaction was allowed to stir at room temperature for 30 min and became cloudy before refluxing for an additional 1 hr. The solution was then cooled to 0 °C, and the ketone (18.1 mmol) was added dropwise in a span of 10 min. The solution was allowed to warm to room temperature and allowed to stir for 2 hours. The reaction mixture was quenched by the addition of 20% aqueous ammonium chloride, the organic layer was separated, and the aqueous layer was extracted with diethyl ether (25 mL × 2). Combined organic layers were washed with saturated sodium bicarbonate (50 mL) and brine (50 mL), dried over $MgSO_4$ and the solvent was evaporated under reduced pressure to afford the crude product. The tertiary alcohol was isolated by flash chromatography (EtOAc/Hexane = 1:6). ^1H NMR (500 MHz, $CDCl_3$) d 8.50 (d, 2H, J = 6.0 Hz), 7.16 (d, 2H, J = 6.0 Hz), 2.75 (s, 2H), 1.46 (s, 1H), 1.23 (s, 6H).

DDL-214c (2-methyl-1-(pyridin-4-yl)propan-2-yl (tert-butoxycarbonyl)alaninate):

In a round bottom flask equipped with a stir bar, the 2-methyl-1-(pyridin-4-yl) propan-2-ol (1.5 mmol) was dissolved with an appropriate amount of dichloromethane to give a 2M concentration of the alcohol. The solution was added to 4-dimethylaminopyridine (DMAP, 1.5 mmol) and the *(tert-butoxycarbonyl)alanine* (3 mmol). The solution was allowed to stir for 10 min at 0 °C before adding N-(3-dimethyl aminopropyl)-N'-ethyl carbodiimide hydrochloride (EDC HO), 3 mmol). The reaction mixture was allowed to warm to room temperature and stirred overnight. The solution was concentrated under vacuum, and the product was isolated by flash chromatography (EtOAc/Hexanes= 1:6). ^1H NMR (500 MHz, $CDCl_3$) d 8.52 (d, 2H, J = 6.1 Hz), 7.14 (d, 2H, J = 6.1 Hz), 4.98 (bd, 1H, J = 6.8 Hz), 4.17 (bm, 1H), 3.09 (d, 1H, J = 13.5 Hz), 3.02 (d, 1H, J = 13.2 Hz), 1.43 (bs, 9H), 1.49 (bm, 6H), 1.27 (d, 3H, J = 7.2 Hz).

DDL-214 (2-methyl-1-(pyridin-4-yl)propan-2-yl alaninate):

In a round bottom flask equipped with a stir bar, the 2-methyl-1-(pyridin-4-yl)propan-2-yl (tert-butoxycarbonyl)alaninate (0.5 mmol) was cooled to 0 °C degrees and was added 1.25 mL of 4M HCl in dioxane and allowed to stir on ice for 1 hr. The solution was allowed to stir for an additional 1 hr at room temperature. The solution was concentrated under a vacuum and was added ether or hexanes. The hydrochloride salt was allowed to precipitate, and the product was filtered and dried. The hydrochloride salt product could also be triturated with diethyl ether. In the case where

the hydrochloride salt product dissolves in ether, the ether was evaporated, and the product was added hexanes and sonicated for 10 min before decanting the hexane solvent and dried under high vacuum to afford DDL-214. ^{1}H NMR (500 MHz, MeOD) d 8.81 (d, 2H, *J* = 4.9 Hz), 8.04 (d, 2H, *J* = 5.8 Hz), 4.08 (q, 1H, *J* = 7.2 Hz), 3.48 (d, 1H, *J* = 13.4 Hz), 3.44 (d, 1H, *J* = 13.3 Hz), 1.62 (s, 3H), 1.59 (s, 3H), 1.48 (d, 3H, *J* = 7.2 Hz). 13C NMR (500 MHz, MeOD) d 169.1, 158.6, 140.9, 129.3, 83.9, 48.8, 46.0, 26.5, 24.61, 24.56, 15.0. HRMS (ESI): calcd for C12H18N2O2 $[M+H]^+$: 223.1441, found: 223.1450.

SFC Separation of DDL-214 enantiomers

The separation of DDL-214 into its two enantiomers was achieved by supercritical fluid chromatography (SFC) performed at Lotus Separations (Princeton, NJ). Briefly, we used a LUX-4 (2 x 25 cm) column and a mobile phase that was 20% ethanol (0.2% NPA)/CO_2, 100 bar, 60 mL/min, injection volume was 0.5 mL, 18 mg/mL in methanol. The chromatogram of the separation is shown in **Figure S2A**. HPLC-SFC separation was accomplished on a 2.169 g scale of DDL-214 enantiomers, yielding 570 mg of DDL-218, and 775 mg of DDL-219. The purity of both DDL-214 enantiomers (DDL-18 and -19) was confirmed by HPLC UV absorbance at 220 nm, confirming both DDL-218 (RT: 4.94 min) and DDL-219 (RT: 6.69 min) to be >99% pure.

HPLC UV spectra of DDL-214, -218 and -219

LUX-4 (25 x 0.46 cm) column was used and a mobile phase that was 20% ethanol (NPA)/CO2, 100 bar, 3 mL/min, 3 mL/min, 220, 254, 280 nm. The purity of both enantiomers was confirmed by HPLC UV absorbance at 220 nm. DDL-218 (RT: 4.94 min) and DDL-219 (RT: 6.69 min) were confirmed to be >99% pure (**Figure S2B and C**, respectively).

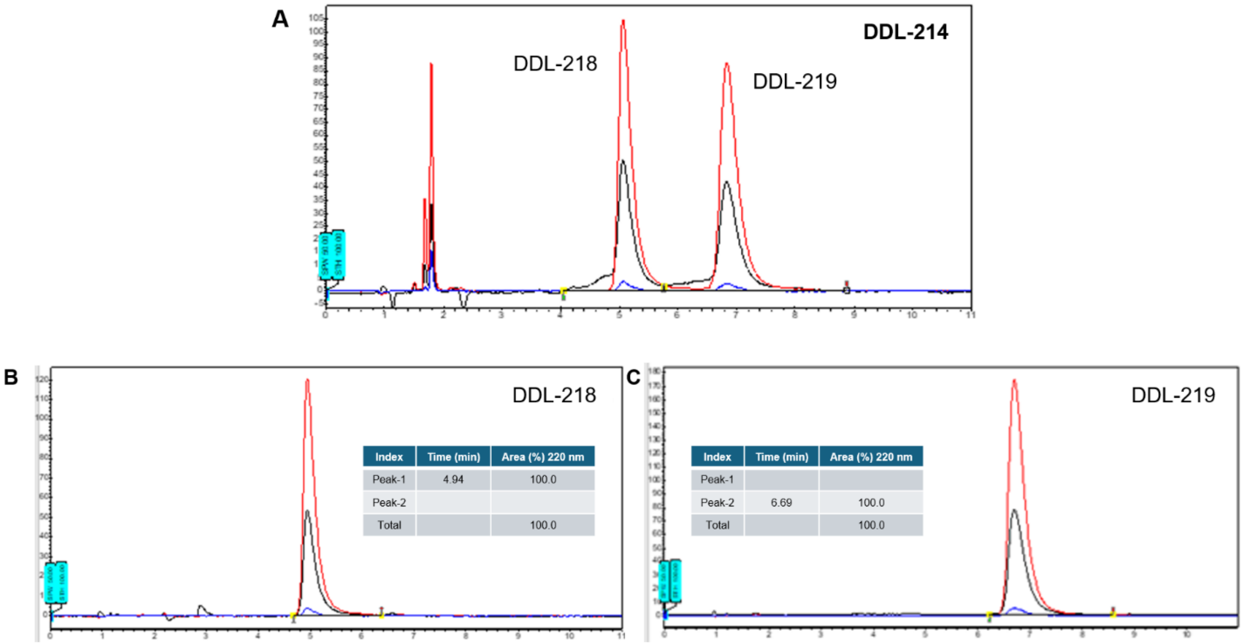

Figure S2. *HPLC-UV chromatograms for DDL-214 enantiomers absorbance at 220, 254 and 280 nm.*

Optical Rotational Activity

Both enantiomers were weighed from the original vial and dissolved in dichloromethane (DCM), with a target concentration of 0.8-1% W/V. A Rudolph Autopol III Automatic Polarimeter was used to assess optical rotational activity.

DDL-218 = 37.5 mg; VDCM = 4 mL (~ 0.94 w/v%)
DDL-219 = 39.5 mg; VDCM = 4 mL (~0.99 w/v%)

After cleaning and zeroing the polarimeter cell, the samples were loaded, and measurements were performed at 25 °C and 589 nm in triplicate. The cell was rinsed between loading samples. The polarimeter performs 3 readings and gives an average, std, min, and max (**Table S1**).

Table S1. Polarimeter Readings

**DDL-218**	**DDL-219**
Ave=-26.33<br>Std=0.47<br>Min=-27<br>Max=-26	Ave=11.67<br>Std=0.47<br>Min=11<br>Max=12
Ave=-28.33<br>Std=0.47<br>Min=-29<br>Max=-28	Ave=10<br>Std=0<br>Min=10<br>Max=10
Ave=-32<br>Std=0<br>Min=-32<br>Max=-32	Ave=10<br>Std=0<br>Min=10<br>Max=10

DDL-218 = -28.9°
DDL-219 = 10.6°

Specific rotation:
Peak 1: $[\alpha]^{25}_{D}$ -28.9° (*c* = 0.94, CH_2Cl_2)
Peak 2: $[\alpha]^{25}_{D}$ +10.6° (*c* = 0.99, CH_2Cl_2)

Figure S3A. *Optical rotation of enantiomers DDL-218 and -219*

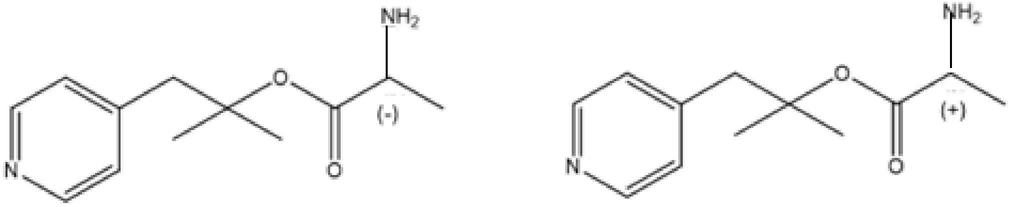

DDL-218 DDL-219

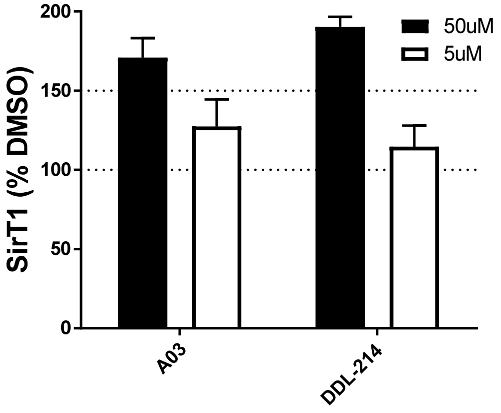

Figure S3B. *SirT1 protein levels **in** N2a-E4 cells after A03 and DDL-214 treatment.* Compound treatment was done at 5 and 50 μM. SirT1 levels were measured by AlphaLISA followed and normalized to protein concentration

DDL-218 Physiochemical Properties

Physiochemical properties of DDL-218 are shown in **Table S2**.

Brain Tissue Binding [fu,bound (%)]

Brain tissue was homogenized in PBS (pH 7.4) (1: 3 weight(mg)/volume(μL)) and the protein concentration of was determined using the Micro BCA™ Protein Assay Kit (Thermo Fisher Scientific, Cat#23235). Brain homogenate was diluted to 20 mg/mL in PBS (pH 7.4) and added to Slide-A-Lyzer™ MINI Dialysis Devices, 10K MWCO dialysis cups (Thermo Fisher Scientific, Cat#PI88401) in 48-well plate containing PBS (500 μL; pH 7.4). 1 μL of 1 mM compound was added to the brain homogenate (Final Concentration: 2 μM compound, 0.5% DMSO) and incubated on a rocker for 4.5 hours at 37 °C. 50 μL of brain homogenate (within the dialysis cup) and PBS (within the 48-well plate) were transferred to new microcentrifuge tubes containing 200 μL of quenching reagent (100% Acetonitrile) containing internal standard. Solutions were clarified by centrifugation (16,000 x g, 5 min) and the supernatants were transferred to new tubes and lyophilized. Samples were reconstituted in 100 μL of 50/50/0.1 (Water/Acetonitrile/Formic Acid) prior to analysis via liquid chromatography-tandem mass spectrometry (LC-MS/MS).

The % of the unbound drug (fu,bound) was calculated using the following equation: fu,bound = [normalized peak area in buffer side / normalized peak area in brain side] x 100

Liver Microsome Stability [t1/2 (min)]

An aliquot (1 μL) of test compound (1 mM, 100% DMSO) was added to an aqueous liver microsome solution (1000 μL, PBS pH 7.4, 0.5 mg/mL human liver microsomes (Thermo Fisher Scientific, Cat# HMMPL), 2 mM NADPH, 2 mM MgCl2) and incubated at 37 °C for 120 min. Aliquots (50 μL) of the microsome solution are taken at various timepoints (0, 5, 10, 15, 30, 60, 90, 120 min) and added to a reaction quenching solution (200 μL 100% Acetonitrile) containing an internal standard. Solutions were clarified by centrifugation (16,000 x g, 5 min) and the

supernatants were transferred to new tubes and lyophilized. Samples were reconstituted in 100 µL of 50/50/0.1 (Water/Acetonitrile/Formic Acid) prior to analysis via liquid chromatography-tandem mass spectrometry (LC-MS/MS).

Normalized chromatographic peak areas was plotted at each time point and the half-life (T1/2) of compound in liver microsomes was determined by using the trendline equation to calculate the time at which compound abundance was 50% of that at timepoint 0 (T0).

Kinetic Solubility

Kinetic solubility was assessed by separately diluting a test compound solution (10 mM, 100% DMSO) into the aqueous buffer (PBS pH 7.4) and DMSO at 100 µM, 100 µL total volume. The solutions were then incubated at 37°C for 90 min and centrifuged (16000xg, 5 min). An aliquot (50 µL) of each supernatant was analyzed by LC-MS/MS.

Five standard concentrations (100, 10, 1, 0.1, and 0, DMSO) in duplicates were incubated at 37°C for 90 min and centrifuged (16000xg, 5 min). Aliquots (50 µL) of supernatant were analyzed by LC-MS/MS to create the standard curve. Kinetic solubility for DDL-218 is 64.4 µM.

**Table S2. DDL-218 Physiochemical Properties**	
MW (g/mol	222.29
Kinetic solubility (µM)	64.4
Brain Tissue Binding [$f_{u,bound}$ (%)]	43
Microsomal stability t1/2 (min)	>140

Affinity purification, STRING analysis, and effects of PRMT over-expression on SirT1

Azide bound DDL-220, an active analog (SirT1 increase of 30% over vehicle at 50 uM in N2a-E4 cells) was used for affinity purification of the proteins from human neuroblastoma SH-SY5Y cells lysates that then underwent proteomics analysis. STRING analysis [1] was then performed, suggesting interaction of the SirT1 enhancer and a PRMT. The column synthesis scheme, silver stain of cell lysate gel, and findings from gel band by LC-MS-MS analysis is shown in **Supplementary Figure S4A-C** respectively.

STRING analysis identified PRMT4 (also known as CARM1, red circle in **Fig. S4C**) as interacting with DDL-214 analog DDL-220, prompting our investigation of PRMTs in overexpression studies to determine their effect on SirT1 expression.

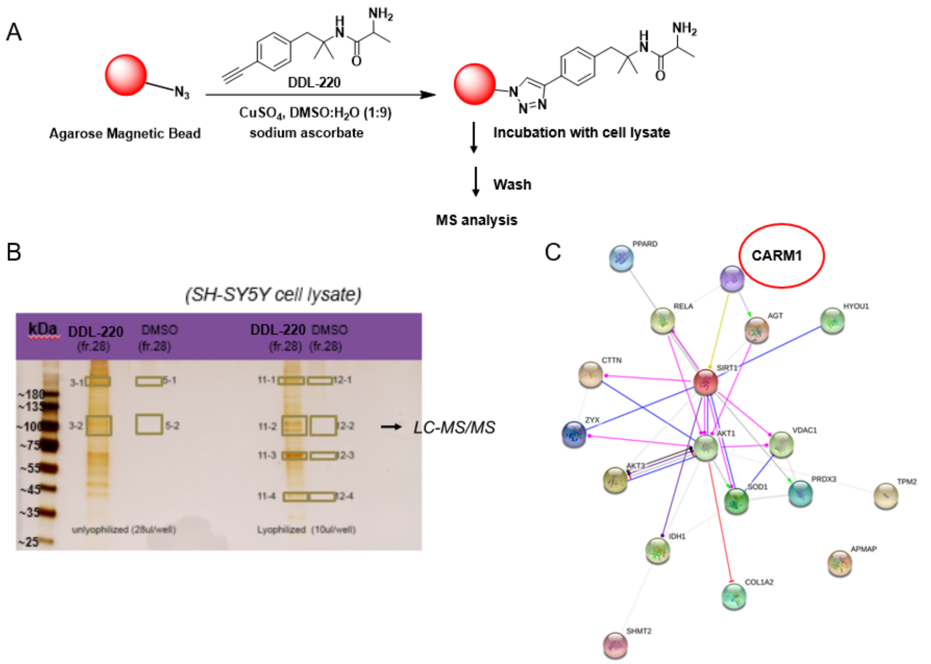

Figure S4. *Synthesis of column, affinity purification, and analysis.* A) Synthesis scheme for coupling of DDL-220 to column azide beads by click coupling. B) Silver stain analysis of cell lysate gel from DDL-220 column extract after affinity purification. C) LC-MS/MS proteomic and STRING analysis reveals multiple proteins including PRMTs (CARM1).

PRMT overexpression

N2a-E4 cells were cultured at 37°C with 5% CO_2 using DMEM media supplemented with FBS and G418. For protein expression, cells (75K/ well in a 24-well plate) were cultured overnight, then transfected with 0.75 µg of PRMT vectors human ORF clone for PRMT1 (Origene # RC224239), PRMT4 (Origene # RC217483), PRMT8 (Origene # RC205188), PRMT5 (Origene # RC203458), PRMT7 (Origene # RC201672) or pCMV6 entry vector (Origene # PS100001) using TurboFectin transfection reagent (Origene# TF81001) for 48 hours following manufacturer's protocol. cells were washed using PBS followed by lysis using 70ul MPER protein extraction reagent (Thermo fisher scientific # 78501) supplemented with protease phosphatase inhibitors (Thermo fisher scientific # 78446). Cells were collected in pre-chilled 1.5ml microcentrifuge tube using a cell scraper and kept on ice for 15mins followed by centrifugation at 14000g for 10min at 4o C. The supernatant was collected and kept at -80 o C until used. Protein concentration was determined by BCA assay.

For immunoblotting, 15-20 µg of protein was ran in a 4-20% Tris-glycine gel (Thermo fisher # XP04205BOX) followed by transfer to a PVDF membrane using BioRad Mini Trans-Blot Electrophoretic Transfer system (60V for 2 h) (BioRad # 1703930). The membrane was probed overnight with primary antibody against protein of interest (**Table 1**) at 4 °C. The next day, following washes with PBST, the membrane was incubated with secondary antibody from LiCOR and scanned using the LiCOR Odyssey infrared scanner. Confirmation of PRMT protein overexpression is shown **in Fig. S5A and B.**

SirT1 levels were measured by AlphaLISA followed and normalized to protein concentration. Briefly, the AlphaLISA was performed using 2 μL antibody. The arbitrary unit (AU) signal was normalized using protein concentration and expressed as fold change with respect to the pCMV6 Entry vector. As shown in **Figure S5C**, PRMT5 elicited the highest fold change in SirT1 protein. PRMT7 did not significantly increase SirT1 **Figure S5E**.

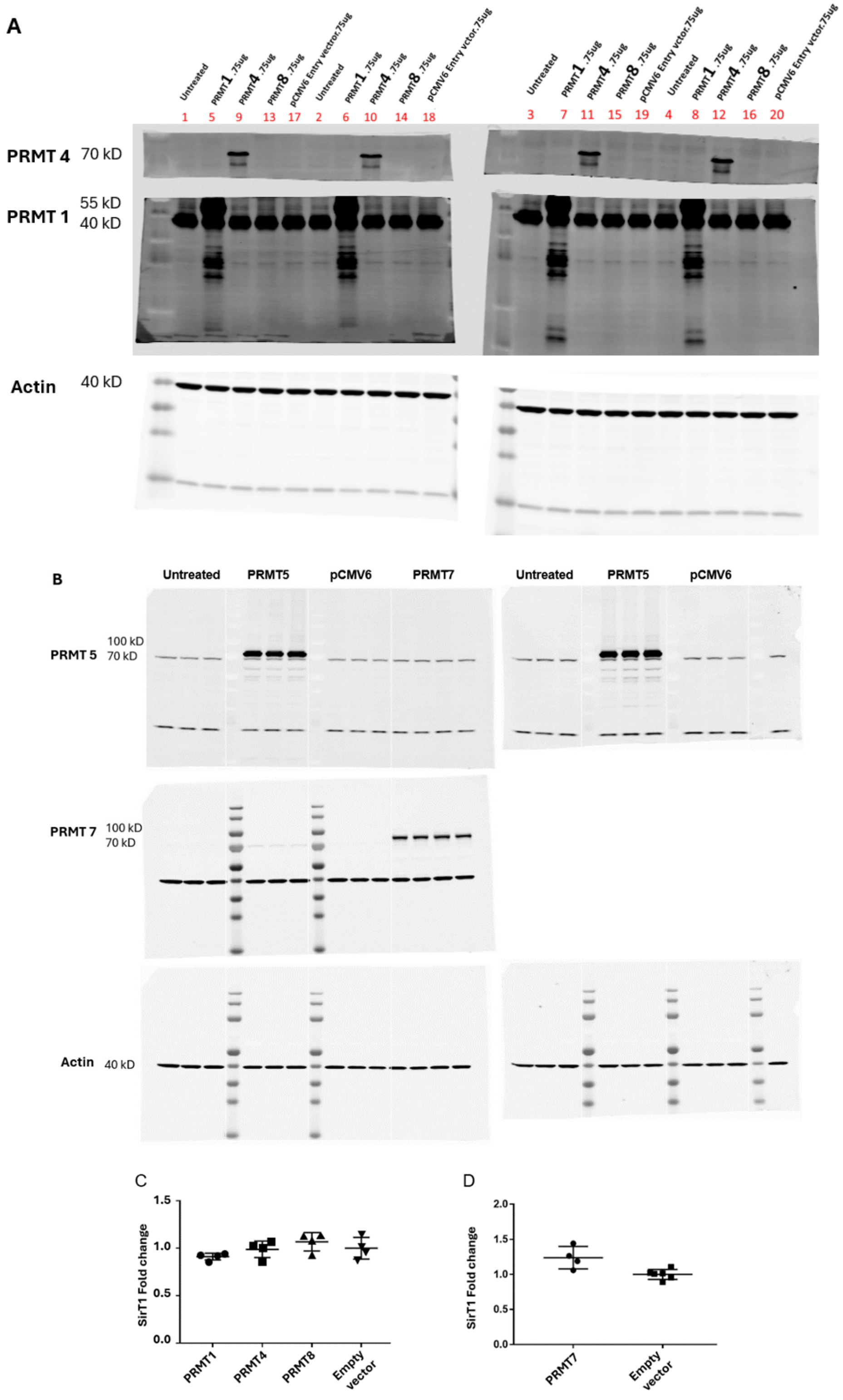

Figure S5. *PRMT over-expression and SirT1 protein levels.* Overexpression of (A) PRMTs1 and 4 in n=4 biological replicates and (B) PRMTs 5 and 7 are shown. SirT1 levels in E4-N2a cells with over-expressed (C) PRMTs 1, 4, and 8 (n = 4); and (D) PRMT 7 (n = 4 for PRMT7; n = 6 for empty vector). Data graphed as the mean and SEM. Statistical analysis performed using an unpaired two-tailed student's t-test : unpaired with Mann Whitney correction where *p < .05 and ***p </= .001.

Correlations between gene mRNAs in DDL-214, -218, and -219-treated Kelly cells

As described in the main manuscript, human Kelly neuroblastoma cells were treated with racemate DDL-214, and enantiomers DDL-218 and -219. Levels of PRMT5, NFYb, SirT1, and PPP2r5e mRNAs were then determined. The correlations between these gene mRNAs for all data points with R^2 values are shown in **Figure S6A-C** below. Data points are graphed by treatment in **Figure S6D-F** and their significant correlation (Pearson correlation 0.914) in bioinformatic analysis of gene co-expression of 2300 human brain RNAseq samples performed at Genefriends.org (Raina, et al. (2022) GeneFriends: gene co-expression databases and tools for humans and model organisms, Nucleic Acids Research, 2022; https://doi.org/10.1093/nar/gkac1031)

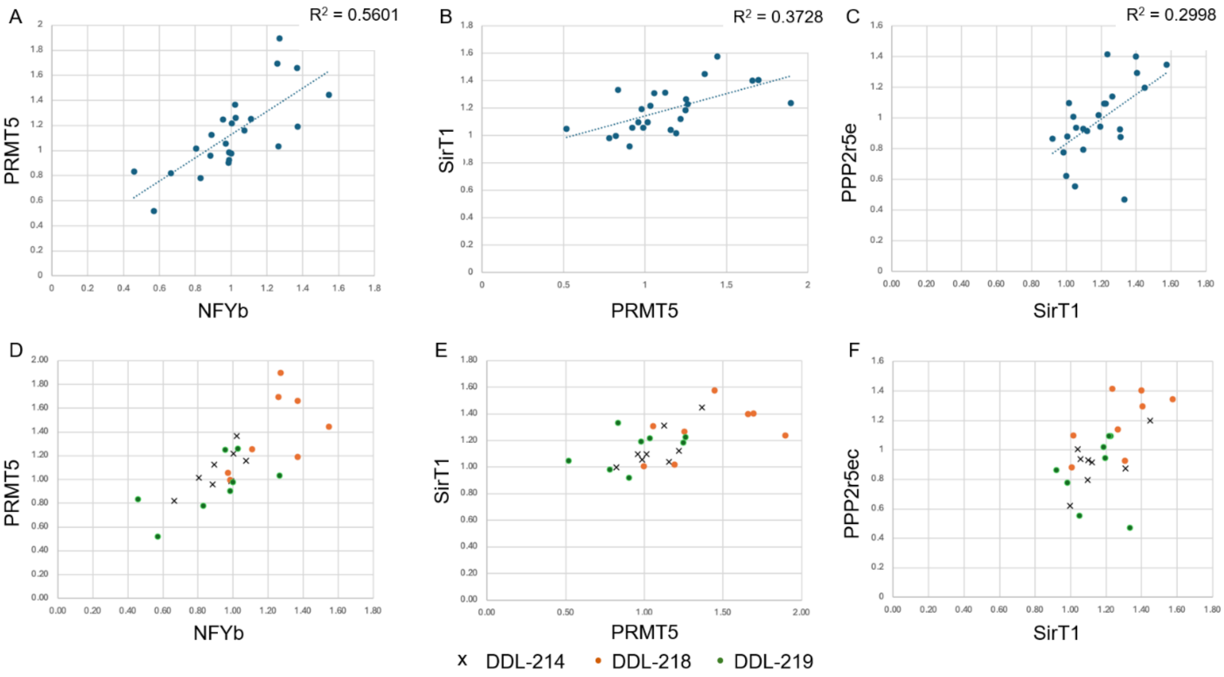

Figure S6. *Correlation of gene mRNA levels in DDL-treated Kelly cells*. All data points are shown for (A) PRMT5 vs. NFYb, (B) SirT1 vs. PRMT5, and (C) PPP2r5E vs. SirT1. R^2 values are shown on the upper right of each panel. (D-F) The same data with treatment (DDL-214, -218, or -219) indicated by symbols shown below panel (E). The statistical 'r' value and significance determined by Pearsons Correlation Coefficient is: PRMT5 vs. NFYb: r=0.7484, p=.000026; SirT1 vs. PRMT5 : r=0.6106, p=.00153; PPP2r5e vs. SirT1: r=0.552, p=.005164.

ChIP assay for SirT1 gene promoter occupancy by ApoE4 and RNA polymerase

In N2a-E4 cells where PRMT5 is overexpressed (**Figure S7**) , the occupancy of the SirT1 gene promoter by ApoE4 or RNA Polymerase (RNAP) was determined using an EZ-ChIP kit (Millipore

17-371) following manufacturer's instructions. Brain was frozen to -80C, then powdered on dry ice and split for either RNA analysis or for the ChIP assay, approximately ~100mg powderized tissue was fixed in formaldehyde. For cultured cells in vitro, cells were fixed in each well and harvested by scraping. Samples were sonicated to break chromatin into 200-1000bp fragments (according to previously optimized conditions), using a Epishear programmable sonicator (Active Motif), and aliquots were subjected to immunoprecipitation with antibodies against either apoE4 (Novus), RNA Polymerase (supplied in the Ez-ChIP kit), or mouse IgG (isotype matched).

Immunoprecipitated chromatin was subjected to conditions that reversed the cross-links, and DNA was purified. The immunoprecipitated DNA was measured for the level of immuno-captured SirT1 gene promoter sequence using qPCR with PCR primers that bind flanking the SirT1 CLEAR sequence. PCR produced an amplicon of 125bp with the CLEAR site near one end, the CLEAR sequence is bold underlined in the following 125bp PCR amplicon:

ACCTCGTCCGCCATCTTCCAACTGCCTCTCTGGCCCTCCGCCCTCGCCTCCTCTGCTCCG
CTCGACGCGCGGCACTGGCGCCCCCGCGGCTCGGCTGCGGGAGATTTAAACCCC**GTCAC**
GTGACC

PCR amplification Ct values for apoE4 and RNAP were normalized to the levels obtained for IgG and further normalized to the level of input DNA. This level of immuno-captured DNA indicates the level of apoE4 or RNAP occupancy of CLEAR site region of the SirT1 gene promoter.

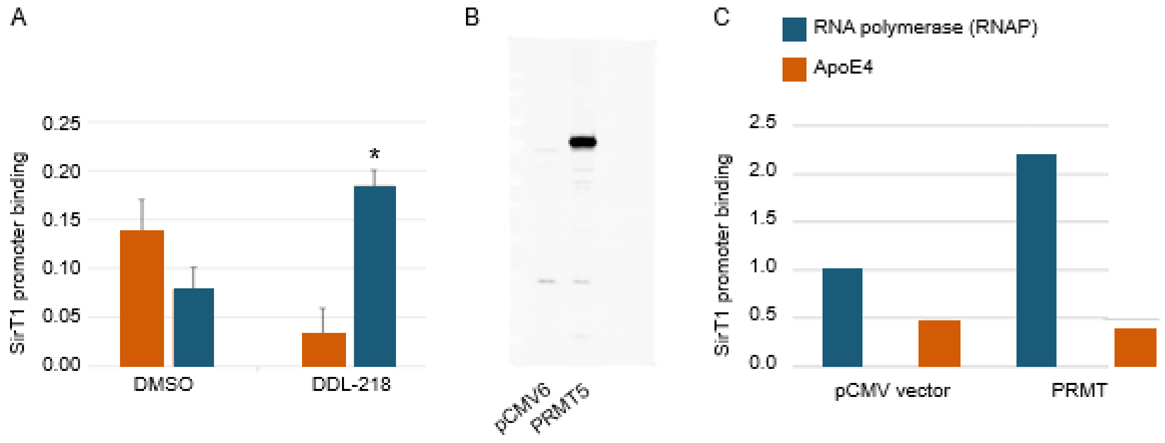

Figure S7. *SirT1 promoter binding by RNA polymerase and ApoE4*. The ChIP assay was used to assess RNA polymerase (RNAP) and ApoE4 occupancy of the SirT1 promoter in Kelly neuronal and N2a-E4 cells. (A) SirT1 promoter binding by either RNA polymerase (RNAP) or ApoE4 in the ChIP assay (n = 2) in samples from Kelly cells treated with either DMSO (vehicle) or DDL-218. Data graphed as mean and SEM. Statistical analysis performed using an unpaired student's t-test where *p<.05. (B) PRMT5 protein in pCMV6 entry vector- or PRMT5 expression vector-transfected N2a-E4 cells. (C) SirT1 promoter binding by RNAP or ApoE4 (n = 1) in samples from N2a-E4 cells transfected with either the control pCMV6 vector or a PRMT5-expressing vector. The legend in C also applies to A.

OCTET assay of ApoE4 – PRMT5/MEP50 interaction

The OCTET Bio-Layer Interferometry (BLI) platform was used to assess ApoE4 – PRMT5/MEP50 interaction and the kinetics of this interaction. MEP50 is a protein cofactor that binds and stabilizes PRMT5, is necessary for activity and recruitment of substrates (**Figure S8**).

Recombinant ApoE2, ApoE3, and ApoE4 (PeproTech catalog #s 350-12, 350-02, and 350-04, respectively) were and biotinylated using NHS-LC-Biotin (100 mol equiv; Thermo Fisher Scientific Cat # 21336) in 50 mM phosphate buffered saline (PBS; pH 6.5) at 4 °C for 24 hours. The number biotin modifications and relative amount of biotinylated ApoE4 was quantified using liquid chromatography-mass spectrometry (LC-MS).

For biomolecular binding kinetics assays on the OCTET platform, OCTET streptavidin (SA) biosensors (catalog # 18-5019, Sartorus) were hydrated in PBS (pH 7.4) for 20 min prior to the start of the assay. The biosensors were then transferred to a 96-well microplate to begin the 5-step kinetics assay, where: 1. Baseline: PBS (pH7.4) for 60 sec; 2. Loading: PBS (pH 7.4) + ApoE2 or ApoE3 or ApoE4 (0.5 ug) for 600 sec; 3.Baseline: PBS (pH7.4) for 60 sec; and 4. Association: PBS (pH 7.4) + analyte (varying concentrations) for 300 sec. The analyte was the recombinant PRMT5/MEP50 Complex (500 nM; Catalog # 31521, Active Motif), PRMT5 (500 nM; Catalog # 31393, Active Motif) and MEP50 (500nM, Cat # QP8020-ec-100ug, Enquire BioReagents). Association (4) was followed by 5. Dissociation: PBS (pH7.4) for 300 sec. Only ApoE4 showed binding to PRMT5/MEP.

The data were processed using the Octet® data analysis suite. Following data correction (Baseline Step Align Y; Association Step Inter-step correction; Savitsky-Golay Filter), a steady state graph was used to calculate the Chi2, R2, Rmax, Rmax Error, Kd and Kd Error for each interaction.

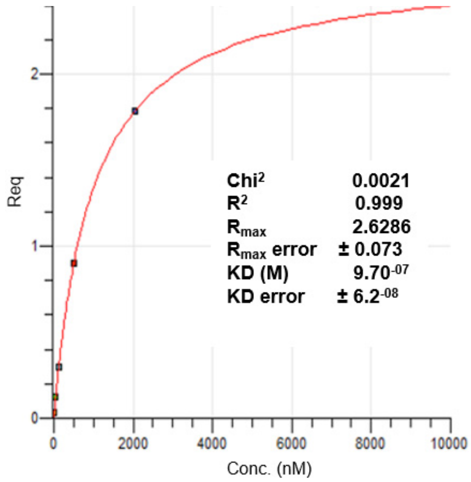

Figure S8. *Curve and values for PRMT5/MEP50:ApoE4 interaction in OCTET*.

Molecular modeling, docking and molecular dynamics simulations

ApoE4 modeling and simulation were used to predict direct binding to coordinated lysosomal expression and regulation (CLEAR) DNA motifs. The ApoE4 structure was modeled from ApoE3 (PDB: 2L7B) [2] by replacing Cys112 to Arg using the mutagenesis tool from PyMol [3]. The mouse CLEAR sequence (GTCACGTGAC) (mCLEAR) as double-stranded B-DNA was built using the nucleic acid builder from AMBER [4]. Prior to model the docking interaction between apoE4 and mCLEAR structures using HEX [5], both ApoE4 and mCLEAR were subjected to energy minimization (5000 steps) with AMBER ff14SB [6] and bsc1 [7] force field, respectively. In HEX, prediction of total energy was based on shape plus electrostatic and 3-D FFT mode. Out of 2000 possible binding modes, the lowest energy conformation of ApoE4:mCLEAR complex was selected for further analysis. To study the effect of asymmetric dimethylation [AD] at Arg112 and the mutation at Arg251 to Gly in the ApoE4, molecular dynamics (MD) simulations were carried out using the AMBER20 program [8] for the ApoE4:mCLEAR, ApoE4Arg112[AD]:mCLEAR and ApoE4Arg251Gly:mCLEAR complexes. We note the asymmetric dimethylation [AD] at Arg112 and the mutation at Arg251 to Gly in the apoE4 were two separate experiments.

The Arg112 to [AD] and Arg251 to Gly were modified using PyMol in the ApoE4:mCLEAR complex. After adding hydrogens, the complex structures were solvated in a truncated octahedral TIP3P box of 12 Å using the AMBER ff14SB, bsc1 and FFPTM [9] force field parameters. The systems were brought to a neutral charge with sodium ions. Periodic boundary conditions, Particle Mesh Ewald summation and SHAKE-enabled 2-femto seconds time steps were used. Langevin dynamics temperature control was employed with a collision rate equal to 1.0 ps−1. A cutoff of 13 Å was used for nonbonding interactions. Initial configurations were subjected to a 1000-step minimization with the harmonic constraints of 10 kcal.mol-1.Å-2 on the heavy atoms in the complex. The systems were gradually heated from 0 to 300 °K over a period of 50 picoseconds (ps) with harmonic constraints. The simulations at 300 °K were then continued for 50 ps during which the harmonic constraints were gradually lifted. The systems were then equilibrated for a period of 500 ps before the 100 nanoseconds (ns) production runs. All simulations were carried out in the NPT ensemble. Equilibration and production run simulations were carried out using the Sander and PMEMD modules (optimized for CUDA) of AMBER20, respectively. All analyses were performed using the cpptraj module of AmberTools 22. The binding free energy for ApoE4, ApoE4- Arg112[AD] and ApoE4-Arg251Gly with mCLEAR were estimated using the MMPBSAGB module in AMBER by taking snapshots (10000) at every 10 ps from the 100 ns production run (**Figure S9 and Suppl. Video1-3**).

Supplementary Video 1: Binding of ApoE4(R112-*DA2*) **to mCLEAR sequence 100ns simulation**

Supplementary Video 2: Binding of ApoE4(R251G) **to mCLEAR sequence 100ns simulation**

Supplementary Video 3: Binding of ApoE4 **to mCLEAR sequence 100ns simulation**

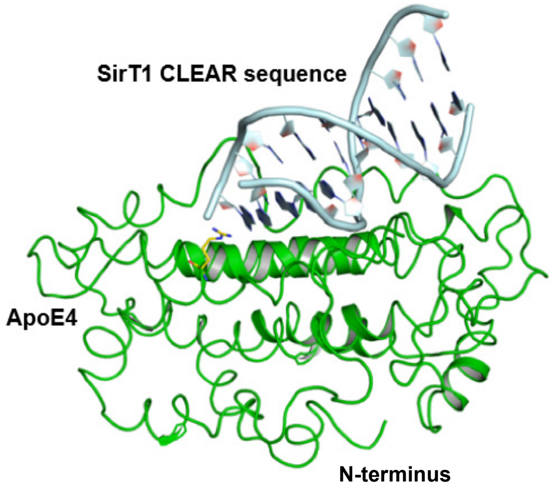

Figure S9. *Molecular modeling of APOE4wt and APOE4-R215G interaction with the SirT1 CLEAR motif*. The modeled interaction of the SirT1 CLEAR sequence (upper) and ApoE4 (lower) is shown. The ΔG for each model is listed.

Expression of ApoE4(R251G) in SH-SY5Y cells

The R251G mutation was incorporated into an expression vector (pCDNA3.1(+) backbone) that expresses apoE4 [10]. DNA was mutagenized using the Q5 Site-directed Mutagenesis Kit (New England Biolabs) and a DNA oligonucleotide primer that incorporates the R251G mutation (CCAGCAGATAgGCCTGCAGGC; lower case "g" is the R251G mutation, rs267606661), according to manufacturer's instructions. Plasmid sequences were verified by Sangar sequencing using primers specific for the GBH region of the pCDNA3 expression vector (Largen).

To assess the effects of ApoE4(R251G) expression in the human SH-SY5Y neuroblastoma cell line, 400K cells were electroporated with APOE4wt or APOE4(R251G) expression plasmids, or the pCDNA3 vector plasmids (0.5 μg DNA) using a Lonza Nucleofector electroporator and electroporation kit (4D Nucleofection Kit with Nucleofection buffer and supplement) (Lonza), according to manufacturer's instructions. Six technical replicate electroporation's were performed over two sessions (n=6). The cells were then plated, cultured for 2 days and harvested for isolation of mRNA (Qiagen RNEasy kit). mRNA was then converted to cDNA (SuperScript™ III First-Strand Synthesis SuperMix kit; ThermoFisher), and SirT1 mRNA levels measured using a qRT-PCR Taqman assay specific for human SirT1 (Thermo Fisher). SirT1 mRNA levels were normalized to GAPDH mRNA levels measured in the same reaction (multiplexing SirT1=VIC and GAPDH=FAM) by the delta-delta-Ct method (**Figure S10**).

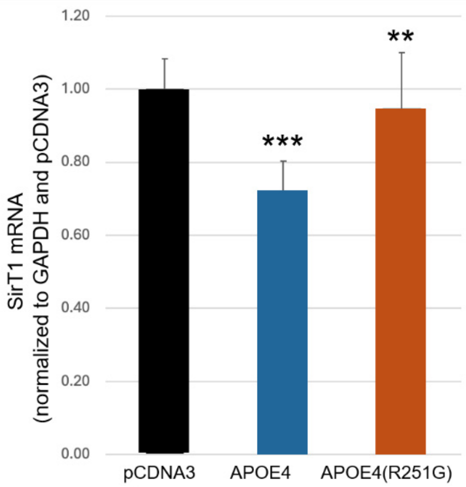

Figure S10. *SirT1 mRNA levels in SH-SY5Y cells electroporated with APOE4, APOE4-R215G, or empty pCDNA3 vector*. SirT1 mRNA was measured (normalized to GAPDH mRNA in the same PCR reaction); levels were normalized to those in the pCDNA3 treated cells (SirT1 mRNA=1.0). (n = X) Data graphed as the mean and SD; n = 6. Statistical analysis performed using one-way ANOVA with Tukey's post-hoc comparisons to pCDNA3 where *(p < .01 and ***p < .001.

Methylation analysis of ApoE4 by PRMT5

To perform methylation analysis, 0.5 µg ApoE4, 0.5 µg ApoE4 + 1 µg PRMT5/MEP50, 0.5 µg histone H4 + 0.5 µg PRMT5/MEP50, 0.5 µg histone H4 + 0.5 µg PRMT5/MEP50 + 1 µM DDL-214, 0.5 µg histone H4 + 0.5 µg PRMT5/MEP50 + 10 µM DDL-214, 0.5 µg histone H4 + 0.5 µg PRMT5/MEP50 + DMSO, or 0.5 µg histone H4 alone. Samples were incubated at 23 ºC for 1 hr. MEP50 is a protein cofactor that binds and stabilizes PRMT5, and is necessary for activity and recruitment of substrates. Samples were then mixed with Laemmli sample buffer supplemented with β-mercapto-ethanol and run on 4-20% gradient gel. The membrane was probed with antibody to monomethylation of arginine (MMA) and anti-ApoE4 at room temperature using the LiCOR Odyssey infrared scanner (**Figure S11**).

Only the histone H4 control (lanes 3-6 and 10-13), and not ApoE4 (lanes 1-2 and 8-9), showed mono-methylation of arginine after incubation with PRMT5/MEP50 without or with DDL-214 or DMSO. Histone H4 without PRMT5/MEP50 was not methylated (lanes 7 and 14).

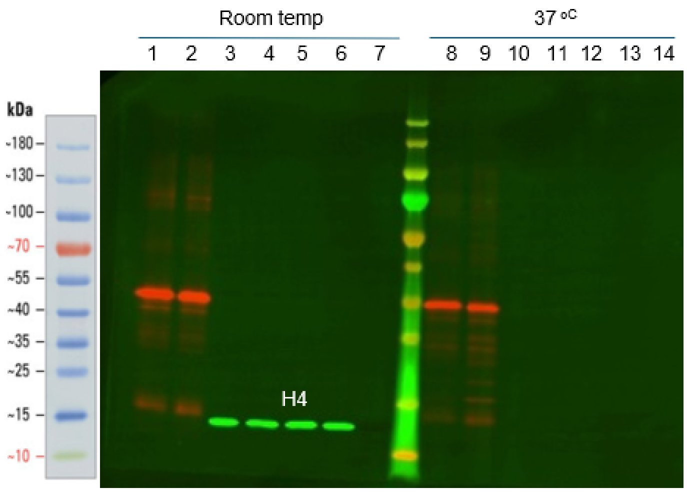

Probe: MMA (Green), ApoE4 (Red)

Lanes
1/8 ApoE4 .5 µg
2/9 ApoE4 .5 µg +PRMT5/MEP50 1 µg
3/10 Histone H4 .5 µg + PRMT5/MEP50 .5 µg
4/11 Histone H4 .5 µg + PRMT5/MEP50 .5 µg + DDL-214 1 µM
5/12 Histone H4 .5 µg + PRMT5/MEP50 .5 µg + DDL-214 10 µM
6/13 Histone H4 .5 µg + PRMT5 .5 µg/MEP50 + DMSO
7/14 Histone H4 .5 µg

Figure S11. *Analysis of methylation*. Gel probed to detect mono-methylation of arginine (MMA; green) and ApoE4 (red) at room temperature (left) and 37°C (right). The samples run on each lane are listed beneath the gels.

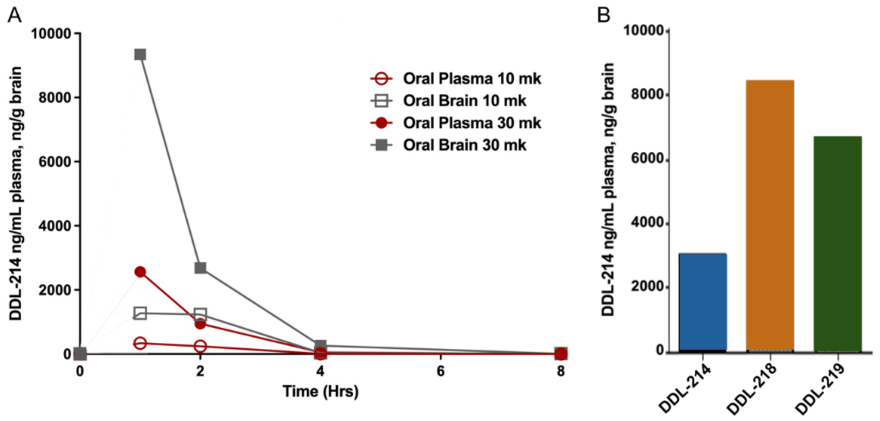

Figure S12. *Pharmacokinetics of DDL-214, -218, and -219 in vivo*. (A) Time course PK study of DDL-214 racemate in mice treated orally at either 10 or 30 mg/Kg, n = 1 per time point. (B) Brain levels of DDL-214,-218 and -219 at the single time point of 1 hour and dose of 30 mg/Kg by oral delivery; n = 1 per condition.

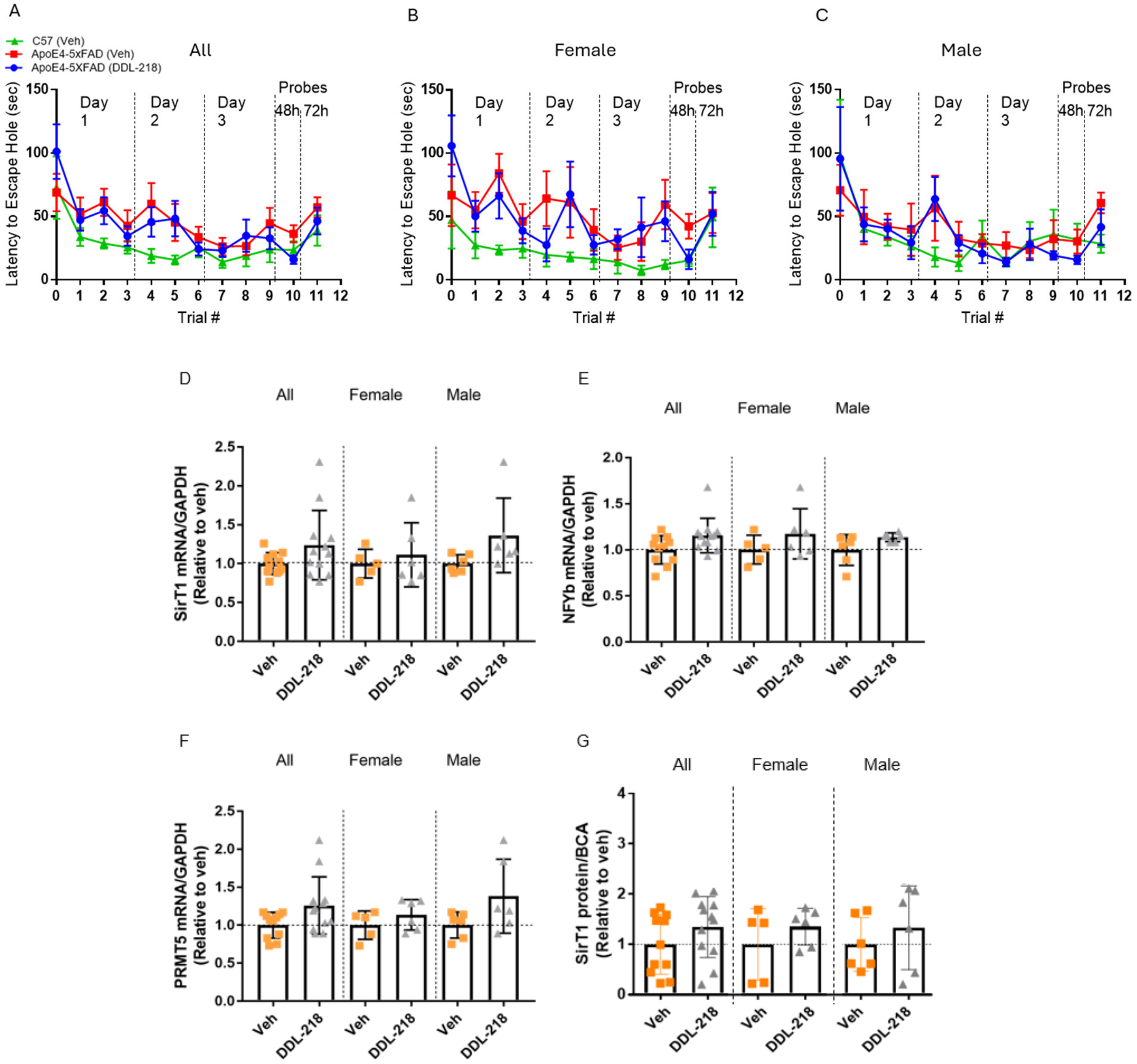

Figure S13. *Barnes Maze latency and brain levels of SirT1, NFYb, and PRT5 mRNA, and SirT1 protein, in females & males, SirT1 protein in brain.* Latency (time to escape hole in seconds, sec) in training and the 48- and 72-hour probes as recorded by ANY-Maze software for (A) All mice, (B) females and (C) males mice in the efficacy study for DDL-218. Levels of (D) SirT1, (E) NFYb, and (F) PRMT5 mRNA. (G) SirT1 protein levels in brain (hippocampus, Hip) for all mice, females, and males. Statistical analysis performed using one-way ANOVA and Tukey-s post-hoc comparison of groups at each trial number for (A) and (B), and a student's t-test for (C) and (D). Differences were not statistically significant.

Global Proteomics

Liquid chromatography tandem mass spectrometry (LC-MS/MS) was used for proteomic analysis. Proteins from hippocampal tissue from mice treated with either DDL-218 or vehicle were diluted in T-PER buffer and concentrations adjusted after BCA protein quantitation assay (Thermo Fisher Scientific). The protein disulfide bonds were reduced and alkylated by treatment with 10 mM tris (2-carboxyethyl) phosphine (TCEP) and 40 mM chloroacetamide (CAA) for 10 min at 95°C. SP3 beads were added to each sample concentrated at 25 µL of beads for 100 µg of protein. Samples were washed 3 times with 2x the sample volume of 80% ethanol in the DynaMag™-2 Magnet and protein solutions diluted 5-fold with 50 mM TEAB. Trypsin was added in a 1:100 (mass trypsin:mass total protein) ratio to target proteins, and the samples were incubated overnight on a rocker at 37°C.

Samples were centrifuged at 16,000 x g for 5 minutes at room temperature. A colorimetric peptide assay (Thermo Fischer Scientific) was run on a sample of each supernatant. The results were used to aliquot 10-mgs of protein from each sample, which were then lyophilized in the SpeedVac. Samples were reconstituted in 100mM TEAB, individually labelled using TMT Isobaric Label Reagent Set (Thermo Fischer Scientific), and incubated at room temperature for one hour. Each sample was then incubated at room temperature for 15 minutes with 5uL of 5% hydroxylamine and lyophilized in the SpeedVac. Subsequently, samples were reconstituted in 2% acetonitrile + 0.1% TFA, and a 5uL aliquot of each sample was pooled together. The pooled sample was desalted with C18 Stagetips made from Empore C18 solid phase extraction disks and lyophilized in the SpeedVac. It was then reconstituted in 2% acetonitrile + 0.1% Formic Acid (FA) and transferred to an autosampler vial. LC/MS2 was performed using a nanoLC and orbitrap mass analyzer using a C18 column and DDA/MS2 mass spectrometer method.

Samples were injected (5 µL) onto a reverse phase nanobore HPLC column (AcuTech Scientific, C18, 1.8 µm particle size, 360 µm x 20 cm, 150 µm ID), equilibrated in solvent E and eluted (500 nL/min) with an increasing concentration of solvent F (acetonitrile/water/FA, 98/2/0.1, v/v/v: min/% F; 0/0, 5/3, 18/7, 74/12, 144/24, 153/27, 162/40, 164/80, 174/80, 176/0, 180/0) using an Eksigent NanoLC-2D system (Sciex (Framingham, MA)). The effluent from the column was directed to a nanospray ionization source connected to a hybrid quadrupole-Orbitrap mass spectrometer (Q Exactive Plus, Thermo Fisher Scientific) operating in the positive ion data-dependent mode alternating between a full scan (m/z 350-1700, automated gain control (AGC) target 3 x 106, 50 ms maximum injection time, FWHM resolution 70,000 at m/z 200) and up to 10 MS/MS scans (quadrupole isolation of charge states ≥ 2, isolation width 0.7 Th) with optimized fragmentation conditions (normalized collision energy of 32, dynamic exclusion of 30 s, AGC target 1 x 105, 100 ms maximum injection time, FWHM resolution 35,000 at m/z 200). The raw data from both analyses was processed using Proteome Discoverer™ (version 2.4). Tryptic peptides containing amino acid sequences unique to individual proteins were used for identification/quantification of each protein.

Raw data was submitted to a database search using Proteome Discoverer 3.1 [ThermoFischer Scientific]. Using the results for TMT labeling efficiency, sample volumes were adjusted to achieve an equal ratio of protein across TMT labeled samples. Remaining samples were pooled in equal ratio, and the pooled sample was desalted and lyophilized in the SpeedVac overnight.

The pooled sample was fractionated using Pierce High pH Reversed-Phase Peptide Fractionation Kit (Thermo Fischer Scientific) according to the manufacturer's protocol. The fractions were dried in the SpeedVac and reconstituted in 0.1% formic acid. Samples were transferred to autosampler vials and LC/MS2 was performed as previously described.

Raw data was submitted to a single database search using Proteome Discoverer 3.1 to determine the identity of the peptides and corresponding proteins. Proteins detected with high confidence by the software were filtered by the p-value of the ratio of the abundance of each protein in samples treated with DDL-218

compared to samples treated with vehicle. Results with an abundance ratio (DDL-218) / (DMSO) p-value greater than 0.05 were eliminated to give 87 proteins with a significantly different abundance in samples treated with DDL-218 compared to DMSO. Of the proteins with a significant abundance ratio p-value of < 0.05 and an abundance ratio above or below the threshold, ten were significantly upregulated by treatment with DDL-218 compared to vehicle (Abundance ratio > 1.2), and one was significantly downregulated (Abundance ratio < 0.8) (**Table S4**). A volcano plot with all proteins detected with high confidence and p-values is shown in **Figure S12A**. This gene set was processed into Enrichr's KEGG 2021 [11] Human Gene Set library.

Table S3. Significantly Altered Proteins Identified by Global Proteomics

Gene	Description	Abundance Ratio (DDL218/Veh)	P-Value	Expression in AD	Biological Function	Ref
Ptprn2	Protein tyrosine phosphatase receptor type N polypeptide 2	1.411	0.04427532	Down	Facilitates vesicle-mediated secretory processes, including insulin secretion in pancreatic beta cells and secretion of neurotransmitters in neuroendocrine cells	12,13
Nubpl	Nucleotide binding protein-like	1.374	0.01756104	N/A	Acts as an Fe-S cluster assembly factor in mitochondrial complex I	14
Cep170	Centrosomal protein 170	1.36	0.03081656	N/A	Promotes microtubule organization during mitosis	15
Scfd2	Sec1 family domain containing 2	1.338	0.01216991	Down	Involved in intracellular vesicle transport and exocytosis	16,17
Fxyd7	FXYD domain-containing ion transport regulator 7	1.317	0.03429047	N/A	Modulates Na+/K+-ATPase activity and may play a role in neuronal excitability	18
Tbc1d17	TBC1 domain family, member 17	1.25	0.04011638	N/A	Functions as a mitochondrial GTPase activator protein and mediates mitophagy [9]	19,20
Rps20	Ribosomal protein S20	1.223	0.01699073	N/A	Forms part of the 40S ribosomal subunit and is involved in protein synthesis [10]	21
Rpl31	Ribosomal protein L31	1.22	0.04043987	N/A	Forms part of the 60S ribosomal subunit and is involved in protein synthesis [11]	22
Shisa6	Shisa family member 6	1.219	0.03297311	Associated mutation	Modulates synaptic transmissions in hippocampal neurons by regulating AMPA-type glutamate receptors [12]	23,24
Luc7l2	LUC7-like 2 (S. cerevisiae)	0.793	0.00939106	N/A	Regulates alternative splicing and innate antiviral response	25

Protein tyrosine phosphatase receptor type N (PTPRN2) (Abundance ratio = 1.411, p-value = .044) was processed into the Enrichr-KG database and the gene set library Gene Ontology (GO) within the database to elucidate the biological functions and pathways associated with the gene (**Figure S14B**). In

addition, other key genes identified as being upregulated were found to be involved in either mitochondrial function (Nubpl and Tbc1d17) or synaptic plasticity (Ptprn2,Scfd2, and Shisa6). Both mitochondrial function and synaptic plasticity are implicated in AD pathogenesis (**Figure S14C**).

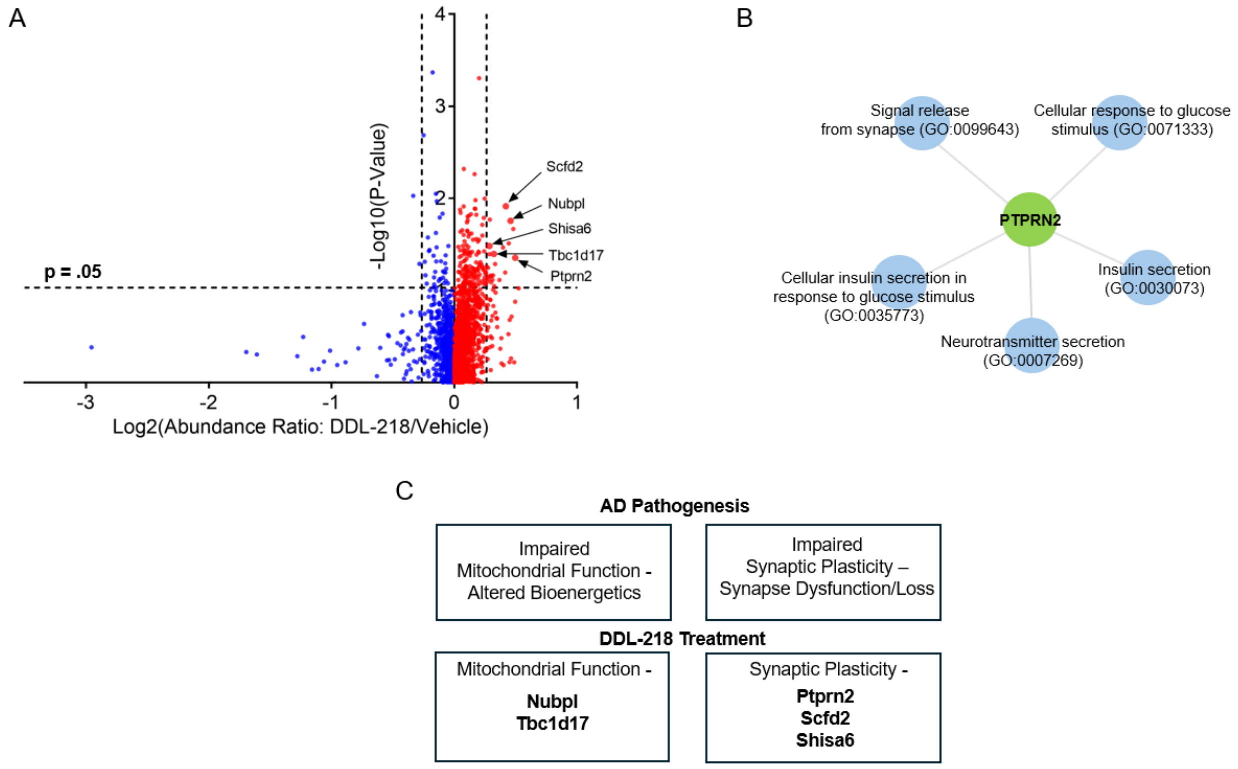

Figure S14. *Proteomics analysis of proteins in brain altered by DDL-218 treatment*. (A) SirT1 protein levels in brain (hippocampus, Hip) in veh and DDL-218-treated ApoE4-5XFAD mice. (B) Volcano plot of the log 2 abundance ratio values for 2518 proteins plotted against their associated -log 10 p-values. The threshold for p = .05 on the y-axis, and the log 2 (0.8) and log 2 (1.2) thresholds on the x-axis, are represented with dashed lines. Key altered proteins Scfd2, Nubpl, Shisa6, Tbc1d17, and Ptprn2 are labeled. (C) Enrichr-KG analysis of processes affected by PTPRN2 utilizing Gene Ontology (GO) Biological Processes gene set is shown. (D) Proteins altered by DDL-218 treatment affect processes, including mitochondrial function and synaptic plasticity, implicated in AD pathogenesis.

NMR and HPLC Analyses

DDL-218: *(-) 2-methyl-1-(pyridin-4-yl)propan-2-yl-alaninate*

^{1}H NMR (500 MHz, $CDCl_3$) δ ppm: 8.52 (br s, 2H), 7.12 (br s, 2H), 3.51 (br s, 1H), 3.06 (s, 2H), 2.49 (br s, 2H), 1.48 (s, 3H), 1.47 (s,3 H), 1.29 (d, *J* = 6.5 Hz, 3H).

^{13}C NMR (125 MHz, $CDCl_3$) δ ppm: 175.3, 149.5, 145.9, 125.9, 82.1, 50.5, 46.1, 26.1, 25.9, 20.3. HRMS-ESI (m/z) $[M+H]^+$ calcd for $C_{12}H_{18}N_2O_2$, 223.14412 found 223.25551.

DDL-219: *(+) 2-methyl-1-(pyridin-4-yl)propan-2-yl-alaninate*

^{1}H NMR (500 MHz, $CDCl_3$) δ ppm: 8.51 (d, *J* = 4.5 Hz, 2H), 7.11 (d, *J* = 6.0 Hz, 2H), 3.54 (q, *J* = 7.0 Hz, 1H), 3.27 (br s, 1H), 3.06 (s, 2H), 1.48 (s, 3H), 1.46 (s, 3H), 1.31 (d, *J* = 7.0 Hz, 3H).

^{13}C NMR (125 MHz, $CDCl_3$) δ ppm: 174.6, 149.5, 145.9, 125.9, 82.3, 50.4, 46.0, 26.1, 25.9, 19.9. HRMS-ESI (m/z) $[M+H]^+$ calcd for $C_{12}H_{18}N_2O_2$, 223.14412 found 223.25451.

DDL-220: *(S)-2-amino-N-(1-(4-ethynylphenyl)-2-methylpropan-2-yl)propenamide*

^{1}H NMR (500 MHz, $CDCl_3$) δ 7.37 (d, 2H, *J* = 8.2 Hz), 7.14 (d, 2H, *J* = 8.3 Hz), 3.76 (q, 1H, *J* = 7.0 Hz), 3.43 (s, 1H), 3.17 (d, 3H, *J* = 13.2 Hz), 3.00 (d, 1H, *J* = 13.2 Hz), 1.41 (d, 3H, *J* = 7.1 Hz), 1.34 (s, 3H), 1.29 (s, 3H).

^{13}C NMR (500 MHz, MeOD) δ 168.7, 138.8, 131.2, 130.3, 120.5, 82.9, 77.1, 54.0, 49.0, 43.5, 26.0, 16.7. HRMS (ESI): calcd for $C_{15}H_{20}N_2O$ $[M+H]^+$: 245.1648, found: 245.1649. Purity estimated by HPLC UV >95%.

HPLC UV spectra of DDL-218

Luna® 3 µm (100 x 0.46 cm) column was used and the method utilized a mixture of solvent A (water) and solvent B (acetonitrile), and the gradient was used for the elution of the compound (min/%B: 0/20, 20/100, 22/50, 30/0). HPLC UV absorbance at 254 nm confirmed the purity of DDL-218. DDL-218 (RT: 11.918 min) was confirmed to be >95% pure.

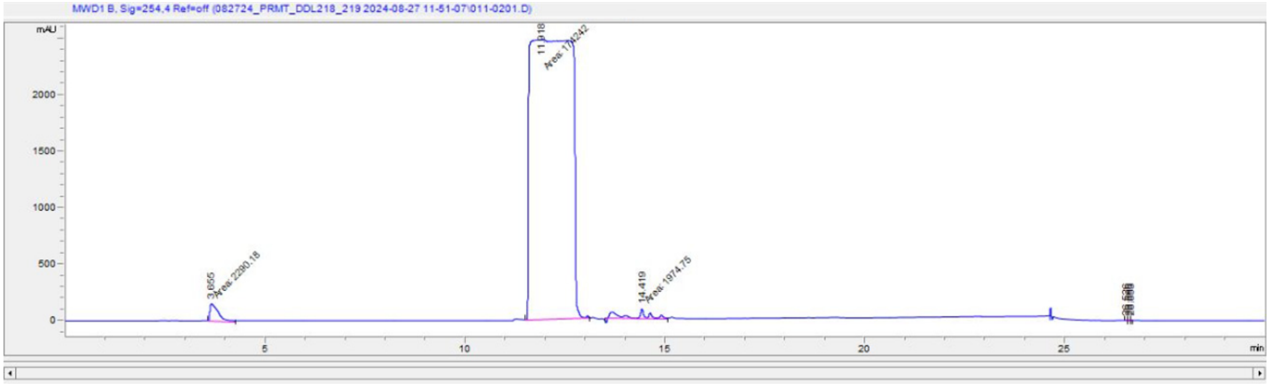

File Information

LC-File	011-0201.D
File Path	C:\Chem32\1\Data\082724_PRMT_DDL218_219 2024-08-27 11-51-
Date	27-Aug-24, 12:25:37
Sample	002_DDL218
Sample Info	
Barcode	
Operator	SYSTEM
Method	032923_30min_c18_4.6mm_analyitical.M
Reference	
Analysis Time	30 min
Sampling Rate	0.0008 min (0.048 sec), 36001 datapoints

#	Time	Area	Height	Width	Area%	Symmetry
1	3.655	2290.2	155.1	0.2461	1.283	0.206
2	11.918	174242	2488.7	1.1669	97.600	0.407
3	14.419	1974.8	85.2	0.3861	1.106	1.407
4	26.526	6.3	2	0.0449	0.004	0.342
5	26.59	6.8	2.2	0.0423	0.004	0.33
6	26.653	6	2.1	0.0379	0.003	0.318

Figure S13. HPLC-UV chromatograms for DDL-218 ((-) 2-methyl-1-(pyridin-4-yl)propan-2-yl-alaninate) absorbance at 254 nm.

HPLC UV spectra of DDL-219

Luna® 3 μm (100 x 0.46 cm) column was used and the method utilized a mixture of solvent A (water) and solvent B (acetonitrile), and the gradient was used for the elution of the compound (min/%B: 0/20, 20/100, 22/50, 30/0). HPLC UV absorbance at 254 nm confirmed the purity of DDL-219. DDL-219 (RT: 12.128 min) was confirmed to be >95% pure.

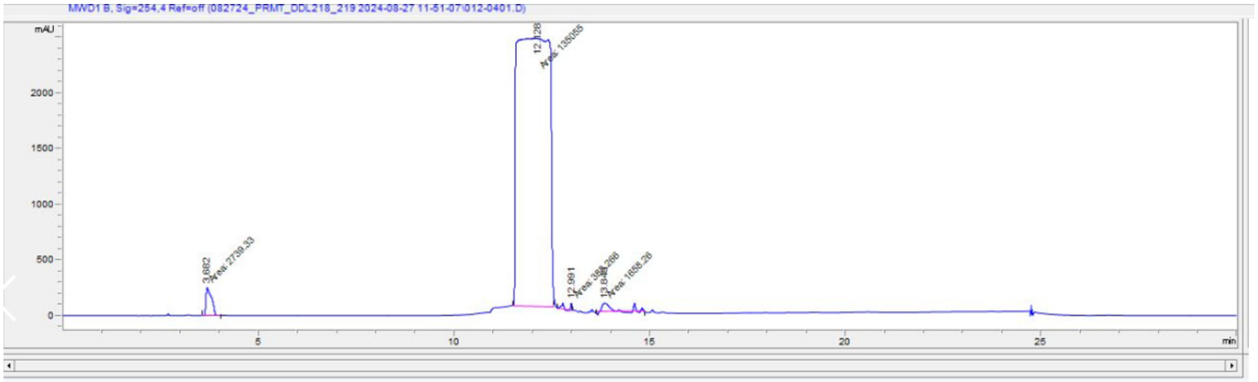

	File Information
LC-File	012-0401.D
File Path	C:\Chem32\1\Data\082724_PRMT_DDL218_219 2024-08-27 11-51-
Date	27-Aug-24, 13:30:40
Sample	004_DDL219
Sample Info	
Barcode	
Operator	SYSTEM
Method	032923_30min_c18_4.6mm_analyitical.M
Reference	
Analysis Time	29.999 min

#	Time	Area	Height	Width	Area%	Symmetry
1	3.682	2739.3	259.2	0.1761	1.959	0.264
2	12.128	135055.3	2409.6	0.9341	96.581	1.582
3	12.991	383.3	72.4	0.0882	0.274	4.335
4	13.845	1658.3	83.7	0.3301	1.186	0.347

Figure S14. *HPLC UV spectra of DDL-219.* HPLC UV absorbance at 254 nm.

Supplementary References

1 STRING, c. Protein-Protein Interaction Networks Functional Enrichment Analysis. *https://string-db.org* (2023).

2 Chen, J., Li, Q. & Wang, J. Topology of human apolipoprotein E3 uniquely regulates its diverse biological functions. *Proceedings of the National Academy of Sciences of the United States of America* **108**, 14813-14818, doi:10.1073/pnas.1106420108 (2011).

3 LLC, S. The PyMOL Molecular Graphics System Version. *PyMOL* **https://pymol.org** (2022).

4 Case, D. A. *et al.* The Amber biomolecular simulation programs. *J Comput Chem* **26**, 1668-1688, doi:10.1002/jcc.20290 (2005).

5 Macindoe, G., Mavridis, L., Venkatraman, V., Devignes, M. D. & Ritchie, D. W. HexServer: an FFT-based protein docking server powered by graphics processors. *Nucleic Acids Res* **38**, W445-449, doi:10.1093/nar/gkq311 (2010).

6 Maier, J. A. *et al.* ff14SB: Improving the Accuracy of Protein Side Chain and Backbone Parameters from ff99SB. *J Chem Theory Comput* **11**, 3696-3713, doi:10.1021/acs.jctc.5b00255 (2015).

7 Ivani, I. *et al.* Parmbsc1: a refined force field for DNA simulations. *Nat Methods* **13**, 55-58, doi:10.1038/nmeth.3658 (2016).

8 Case, D. A. *et al.* AMBER 2020. *Ambertools* **www.ambermd.org** (2020).

9 Khoury, G. A., Thompson, J. P., Smadbeck, J., Kieslich, C. A. & Floudas, C. A. Forcefield_PTM: Ab Initio Charge and AMBER Forcefield Parameters for Frequently Occurring Post-Translational Modifications. *J Chem Theory Comput* **9**, 5653-5674, doi:10.1021/ct400556v (2013).

10 Chang, S. *et al.* Lipid- and receptor-binding regions of apolipoprotein E4 fragments act in concert to cause mitochondrial dysfunction and neurotoxicity. *Proceedings of the National Academy of Sciences of the United States of America* **102**, 18694-18699, doi:10.1073/pnas.0508254102 (2005).

11 Lab, M. a. Enrichr KEGG. *https://maayanlab.cloud/enrichr-kg* (2021).

12 Hokama M et al. *Cerebral Cortex* **24**, 2476-2488, doi:10.1093/cercor/bht101 (2014).

13 Park, J.-H. *et al.* Novel Alzheimer's disease risk variants identified based on whole-genome sequencing of APOE ε4 carriers. *Translational Psychiatry* **11**, 296, doi:10.1038/s41398-021-01412-9 (2021).

14 Medicine, N. L. o. NUBPL NUBP iron-sulfur cluster assembly factor, mitochondrial [Homo sapiens (human)]. *National Center for Biotechnology Information* **https://www.ncbi.nlm.nih.gov/gene/80224** (2024).

15 Medicine, N. L. o. CEP170 centrosomal protein 170 [Homo sapiens (human)]. *National Center for Biotechnology Information* **https://www.ncbi.nlm.nih.gov/gene/9859** (2024).

16 Medicine, N. L. o. SCFD2 sec1 family domain containing 2 [Homo sapiens (human)]. *National Center for Biotechnology Information* **https://www.ncbi.nlm.nih.gov/gene/152579** (2024).

17 Medicine, N. L. o. Application of biomarker SCFD2 in Alzheimer's disease treatment (patent). *National Center for Biotechnology Information* **https://pubchem.ncbi.nlm.nih.gov/patent/CN-109182513-B** (2024).

18 Béguin, P. *et al.* FXYD7 is a brain-specific regulator of Na,K-ATPase α1–β isozymes. *The EMBO Journal* **21**, 3264-3273-3273, doi:https://doi.org/10.1093/emboj/cdf330 (2002).

19 Medicine, N. L. o. TBC1D17 TBC1 domain family member 17 [Homo sapiens (human)]. *National Center for Biotechnology Information* **https://www.ncbi.nlm.nih.gov/gene/79735** (2024).

20 Yamano, K., Fogel, A. I., Wang, C., van der Bliek, A. M. & Youle, R. J. Mitochondrial Rab GAPs govern autophagosome biogenesis during mitophagy. *Elife* **3**, e01612, doi:10.7554/eLife.01612 (2014).
21 Medicine, N. L. o. RPS20 - ribosomal protein S20. *National Center for Biotechnology Information* **https://www.ncbi.nlm.nih.gov/gene/6224/ortholog/?scope=7742** (2024).
22 Medicine, N. L. o. RPL31 ribosomal protein L31 [Homo sapiens (human)]. *National Center for Biotechnology Information* **https://www.ncbi.nlm.nih.gov/gene/6160** (2024).
23 Medicine, N. L. o. SHISA6 shisa family member 6 [Homo sapiens (human)]. *National Center for Biotechnology Information* **https://www.ncbi.nlm.nih.gov/gene/388336** (2024).
24 Ramos, J. *et al.* Genetic variants in the SHISA6 gene are associated with delayed cognitive impairment in two family datasets. *Alzheimer's & Dementia* **19**, 611-620, doi:https://doi.org/10.1002/alz.12686 (2023).
25 Chen Li, et al., The RNA-binding protein LUC7L2 mediates MITA/STING intron retention to negatively regulate innate antiviral response. *Cell Discovery* **7***,* 46 https://doi.org/10.1038/s41421-021-00277-y (2021).
